# Visualizing and Quantifying Impact with Mechanochromic Sensing Paints Based on Self‐Assembled Polydiacetylene‐Silk Core‐Shell Vesicles

**DOI:** 10.1002/advs.202518144

**Published:** 2026-01-04

**Authors:** Marco Lo Presti, Giulia Guidetti, Elisabetta Ruggeri, Terri Lyne Carrington, Fiorenzo G. Omenetto

**Affiliations:** ^1^ Silklab Department of Biomedical Engineering Tufts University Medford Massachusetts USA; ^2^ Berklee College of Music Boston Massachusetts USA; ^3^ Department of Physics Tufts University Medford Massachusetts USA; ^4^ Department of Electrical and Computer Engineering Tufts University Medford Massachusetts USA

**Keywords:** core–shell vesicles, force sensor, impact sensor, mechanochromic sensors, polydiacetylene, responsive coatings, silk fibroin

## Abstract

Rapid detection and quantification of mechanical impact on surfaces is relevant for many fields, as it enables monitoring of material stresses and integrity or to assess the physiological effects of force on humans for concussions, gait, or blast dosimetry, among others. Accurate and quantitative detection of impact force, especially at high values (100–770 N), is challenging and often relies on electronic transduction and interfaces, with associated limitations in integration, shaping, and scalability. We present here a mechanochromic paint as a simple alternative for impact detection and visualization. The paint is based on the self‐assembly of polydiacetylene (PDA) and regenerated silk fibroin protein (SF) into core‐shell vesicles, which exhibit an irreversible mechanochromic transition from blue to red upon impact. The paint enables the tracking of the impact history on the applied material with a colorimetric response that is directly proportional to the impact energy across a broad sensing range of 100–770 N (corresponding to 100–1000 MPa) and has wide utility given by its ability to be interfaced or integrated in a wide variety of material formats, as illustrated by through several demonstrator devices.

## Graphical abstract

Tracking physical impacts is important in many fields. Self‐assembled microparticles made from polydiacetylene and silk fibroin that change color from blue to red when hit can provide an alternative approach to traditional mechanical transducers, quantitatively visualizing impact with responses ranging from <100 to 770 N. The sensors are wireless, stable, scalable, and flexible in design, making them useful for small‐ and large‐scale mechanical monitoring.

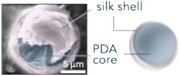

## 1 Introduction

Unlike commercially available mechanical sensors that rely on electronic systems with additional circuitry and displays [1], colorimetric mechanical sensors offer distinct advantages. These include the ability to be applied on large and irregular surfaces, being lightweight, cost‐effective, and requiring no power for continuous monitoring and distributed sensing [2]. As a result, they are particularly useful as impact and damage indicators in sports equipment, household products, smart packaging, and structural material monitoring [3].

Colorimetric sensors can be designed to be either reversible or irreversible. Reversible sensors, such as those based on spiropyran mechanochromic molecules [4], can be used multiple times but do not retain a history of the impacts, as their structural and color changes are reversible [5, 6, 7]. Irreversible sensors, on the other hand, offer a single‐use functionality that records the impact history a material has been subjected to, marking the mechanical force applied [8]. One approach to fabricating such sensors involves encapsulating mechanochromic molecules within a second polymer, which preserves the colorimetric response of the sensing element while providing chemical and mechanical protection, thus enhancing the sensor's selectivity and durability [9].

Polydiacetylenes (PDA) polymers have been extensively studied for sensing applications due to their versatile structural and spectral features [10]. More specifically, they can undergo a blue‐to‐red colorimetric transition as a response to several external stimuli including solvents [11], temperature [12], mechanical stress [13, 14], light [15], pH [16], metallic ions [17], anions [17], surfactants [18], microorganisms [19], and biomolecules [20], thus offering a number of opportunities for developing biological and chemical sensors. Usually, PDAs show an absorption peak at ∼ λ_blue phase_ = 640 nm, which appears visually as an intense blue color that, upon interaction with external stimuli, shifts hypsochromically to ∼ λ_red phase_ = 540 nm, which appears as a red color easily detectable by the naked eye.

Mechanochromism in PDAs occurs when mechanical energy is applied to the polymer, causing internal rearrangement and triggering the color change [21, 22].

**FIGURE 1:**
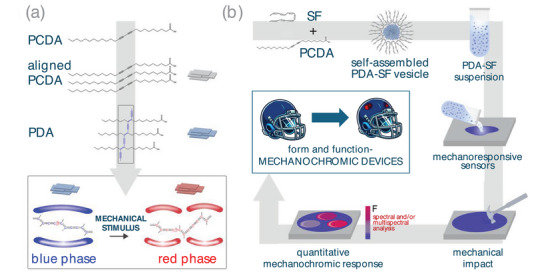
(a) Schematic representation of the chemical synthesis of mechanochromic PDA molecules starting from PCDA. Upon UV activation, the aligned PCDA exhibits a color transition from transparent to blue, while upon mechanical stimulation, the PDA exhibits a mechanochromic transition from blue (blue phase) to red (red phase) due to the rearrangement of the molecular orbitals (as shown in the inset). (b) Schematic representation of the fabrication of polydiacetylene‐silk fibroin (PDA‐SF) mechanochromic sensors. The SF solution and the solid PCDA are mixed to induce the self‐assembly of PDA‐SF micrometer‐size vesicles that can be cast on solid substrates to form mechanoresponsive sensors. A blue‐to‐red color transition occurs upon mechanical impact with the intensity of the transition being directly proportional to the impact force. The PDA‐SF sensors can be conformally applied in various ways to a variety of surfaces and display a color change visible to the naked eye.

The molecular dynamics underlying mechanochromism and the color transition in polydiacetylenes have been explained by the topochemical polymerization of diacetylene monomers, which creates an extended conjugated ene–yne backbone. In its ordered, planar conformation, the conjugated π‐electron system extends along the polymer backbone, producing a strong absorption around 640–660 nm (blue phase). Upon exposure to external stimuli such as mechanical stress and/or torsional strain, conformational changes are introduced into the backbone. These distortions shorten the effective conjugation length, and the resulting disruption of planarity leads to a hypsochromic shift toward shorter wavelengths (∼540–560 nm), observed as a blue‐to‐red color transition (Figure 1a) [10]. While this property has been studied [23], relatively few reports focus on the quantification of mechanical energy needed for the color transition [24], and the existing studies typically address only low‐force regimes such as nanoscale perturbations, weak friction, or soft compression [10, 25, 26, 27, 28].

More specifically, recent literature has increasingly focused on the colorimetric detection of progressively smaller forces, ranging from the newton scale [26] to fractions of a newton [29], down to the millinewton [30] and even nanoscale regimes [31] as summarized in Table S1.

PDA‐based sensors are commonly synthesized as self‐assembled bilayer liposomes by the combination of 10–12 pentacosadyenoic acid (PCDA) and the phospholipid 1,2‐dimyristoyl‐sn‐glycero‐3‐phosphocholine (DMPC) [32, 33, 34, 35, 36, 37]. Their preparation requires first a self‐alignment of the diacetylene monomer that polymerizes through the 1,4‐addition by UV exposure, which generates the mechanoresponsive polymer.

Regenerated Silk fibroin (SF), a structural protein derived from Bombyx mori cocoons, is widely used due to its favorable mechanical, chemical, and optical properties [38]. Its tunable conformation by directed assembly enables the creation of functional materials [39, 40, 41] suitable for optics [42, 43, 44], electronics [45], and sensing applications [46, 47, 48, 49]. Additionally, SF's biocompatibility and ability to stabilize sensitive compounds have made it popular in the biomedical field [50, 51, 52].

SF's primary sequence consists of alternating hydrophobic and hydrophilic regions [53] that confer the ability to self‐assemble into clear vesicular structures within aqueous solutions [54].

In this work, the ability of silk to form micellar structures [54, 55] is leveraged to create cost‐effective, self‐assembled micrometer‐sized core‐shell vesicles with a mechanochromic PDA core and a protective silk shell, designed to function as colorimetric impact sensors (Figure 1b). Unlike recent works that rely on planar architectures [26, 29, 30, 31, 56, 57, 58, 59], here we propose a 3D organization assembled into spherical particles, which can be readily applied onto surfaces with different chemistries and roughness. These vesicles can be applied onto paper substrates, where they exhibit a visible colorimetric transition in response to impact forces starting from 220 N. The detection range explored (F = 220–440 N) is the highest and widest reported for colorimetric mechanochromic sensors detectable by bright‐field imaging [8, 60, 61]. We attribute the markedly higher activation threshold of PDA–SF vesicles to the elastic buffering and mechanical confinement imposed by the stiff, β‐sheet–rich silk shell, which absorbs low‐level impacts and amplifies transmitted stresses only at higher loads, consistent with mechanophore behavior in confined polymer architectures [62]. This range can be further extended to 110–770 N when using fluorescence analysis. Representative demonstrator devices were fabricated to illustrate the versatility of the PDA‐SF vesicles by applying them onto different surfaces and substrates. These prototypes highlight the potential broad utility of the vesicles for various devices, demonstrating their suitability for mechanical monitoring across multiple application domains. This simple approach demonstrates the potential for wide‐ranging applications in detecting mechanical forces using simple, visual cues.

## 2 Results and Discussion

Micro‐colorimetric mechanical sensors were synthesized via a scalable self‐assembly process that involved tip sonication of an aqueous solution containing SF and PCDA, followed by centrifugation (Figure S1). This procedure resulted in the formation of a cloudy suspension of vesicles composed of both PCDA and SF as confirmed by the presence of characteristic Fourier‐Transform Infrared Spectroscopy (FTIR) peaks for both materials (see Figure S2 for peak attribution).

**FIGURE 2:**
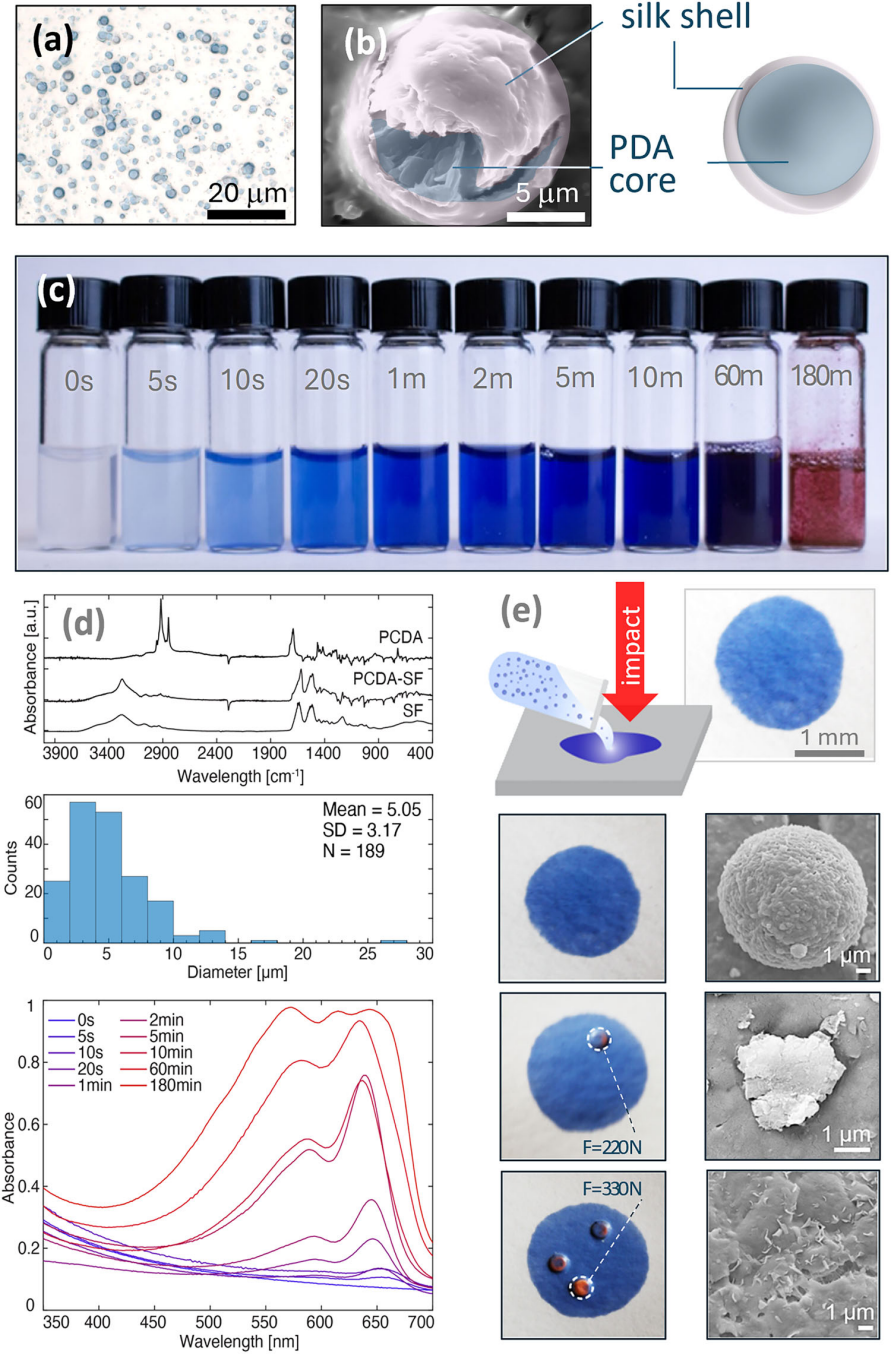
(a) Microscope image of PDA‐SF vesicles and (b) Top‐view SEM image of a PDA‐SF vesicle showing the core–shell structure with PDA sheets in the core of the vesicle and a surrounding conformal silk layer forming the shell. (c) Images of PDA‐SF solutions that are UV‐activated with exposures varying from seconds to hours. (d) From top to bottom: (top) FTIR spectra of PCDA, PCDA‐SF, and SF dried films. (middle) Size distribution of PDA‐SF vesicles with reported mean diameter (Mean), standard deviation (SD), and number of analyzed particles (N) (bottom). PDA‐SF solutions absorbance spectra as a function of wavelength for UV activation times of 0 s–180 min. (e) Application of the PDA‐SF solution on paper. Macroscopic pictures of PDA‐SF paper sensors and corresponding SEM images of the PDA‐SF vesicles before indentation (top), indented with a force of F = 220 N (middle, dashed line), and indented with F = 330 N (bottom, dashed line). [Correction added on 20 January 2026 after online publication: figure 2 is updated.]

The fabricated vesicles have a mean diameter of (5.1 ± 3.2) µm (mean ± SD, N = 189, Figure 2a,d); morphologically, the crystalline PDA sheets [63, 64] assemble in a dense core, while SF forms a continuous layer around them forming the core‐shell structure, as confirmed by scanning electron microscopy (SEM) (Figure 2b). The size of the PDA sheets and the resulting average diameter of the core–shell particles are consistent with other layered PDA nanomaterials reported in the literature [26, 65], here presented in the form of compacted individual microspheres. The core‐shell assembly is further supported by elemental analysis that shows a higher content of nitrogen in the silk shell compared to the core (Figure S3). For comparison, PDA‐DMPC vesicles of a similar size were fabricated following the same protocol (Experimental Section). However, no core‐shell structure is observed, and the surface morphology appeared heterogeneous with exposed PDA sheets (Figure S4).

The PDA‐SF vesicles exhibit higher absorbance intensities (Figure 2d) compared to PDA‐DMPC vesicles (Figure S5), suggesting either a greater PCDA loading into the micellar structure or a more effective crystallization of the diacetylenes. Both hypotheses seem plausible due to silk's amphiphilic structure and its high β‐sheet content, which has been shown to have a templating effect on the diacetylene crystallization process [66, 67, 68]. Additionally, the PDA‐SF suspension demonstrated greater stability (η_PDA‐SF_ = – 47.70 mV) than the PDA‐DMPC suspension (η_PDA‐DMPC_ = – 21.88 mV) which formed macroscopic aggregates a few days after synthesis.

Despite the fact that UV activation of PCDA into PDA is a necessary step to trigger the mechanosensitivity of the polymer (blue phase), prolonged UV exposure is known to induce an irreversible blue‐to‐red transition (red phase) in PDAs, rendering them ineffective as colorimetric sensors [63, 69]. Thus, the effect of UV exposure time on the polymerization and consequent degradation of PDA‐SF is investigated in liquid (Figure 2c) and solid (Figure 2d, Figures S6 and S7) formats. The results were compared to PDA‐DMPC vesicles to demonstrate the enhanced resilience and stability of PDA‐SF (Figures S5 and S6).

In solution, exposure to UV light at λ = 254 nm for t ≥ 5 s activates the blue phase of PCDA, as confirmed by the characteristic absorbance peak at λ_blue phase_ = 640 nm [70] (Figure 2d) and by the corresponding macroscopic pictures of the PDA‐SF suspensions (Figure 2c); for t ≥ 2 min the PDA‐SF red phase starts to form as shown by the increased absorbance at λ_red phase_ = 540 nm [63, 69]; the blue phase is completely degraded for exposure times of t ≥ 180 min. By comparing the activation kinetics of the PDA‐SF suspension with that of PDA‐DMPC (Figure S5), it can be noted that the PDA‐SF suspension displays a much higher absorption in the blue region for the same activation time (PDA‐SF 0.48 a.u. *vs* PDA‐DMPC 0.21 a.u. at t = 2 min), thus suggesting a higher packing efficiency of PCDA within SF‐based sensors [71]. Also, for PDA‐DMPC solutions, the blue‐to‐red transition is complete after just 10 min of UV exposure. The analysis demonstrates that PDA‐SF exhibits greater resistance to prolonged UV exposure, maintaining its mechanosensitivity and delaying the irreversible blue‐to‐red transition, whereas PDA‐DMPC vesicles were more susceptible to degradation under the same conditions. This underscores the importance of the silk fibroin, which provides additional stability and protection to the PDA core in the UV region [72], limiting cross‐sensitivity and extending the lifespan of PDA‐SF when exposed to sunlight. These characteristics make PDA‐SF a viable and robust option for colorimetric sensing applications, especially in environments subject to UV exposure.

Applying the same suspensions of PDA‐SF (Figure 2) and PDA‐DMPC vesicles on paper produces results consistent with those observed in liquid, as confirmed by reflectance measurements (Figure S6).

From the macroscopic photographs of PDA‐SF sensors, it can be observed that PDA‐SF vesicles begin their transition to red only after receiving a UV dose of 1500 mJ/cm^2^ (Table S2), indicating excellent sensor stability against non‐specific stimuli like sunlight. In contrast, PDA‐DMPC vesicles (Figures S6 and S7) start their red transition after just 150 mJ/cm^2^ of UV exposure. Moreover, a simple comparison of the two photographic images reveals that the PDA‐SF system achieves an improved and more homogeneous deposition.

The process described in this study is scalable, allowing for the synthesis of gram‐scale quantities of the sensor. In contrast, existing literature mainly reports the production of PDA‐based sensors on a smaller scale, typically in the range of tens of milligrams [73].

**FIGURE 3:**
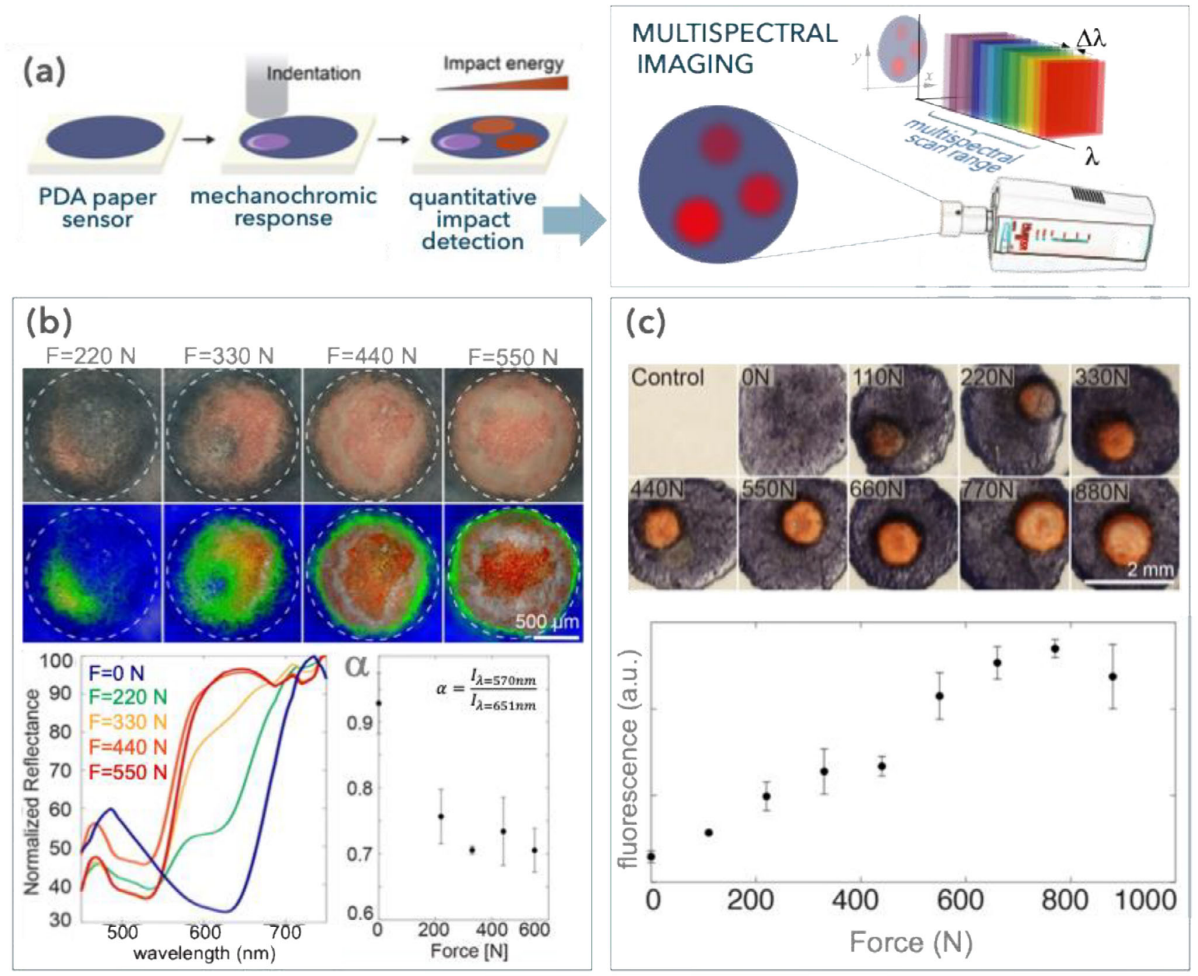
(a) Schematic of the quantification of mechanochromic sensors on paper. The PDA‐SF ink is deposited onto a paper substrate and impacted with controlled force by a projectile causing indentations with different impact forces. Imaging of the paper sensor is done with a multispectral camera across the visible range. (b) Multispectral analysis of PDA‐SF paper‐based sensors showing the original bright field reflection micrograph (top row) and the corresponding reconstructed false‐color image (bottom row) as a function of indentation force (F = 220–550 N). The colors represented in the reconstructed images exemplify the impact distribution in the sensors: blue – substrate F = 0 N, green – F = 220 N, yellow – F = 330 N, orange – F = 440 N, and red – F = 550 N. The corresponding normalized reflectance spectra for PDA‐SF sensors as a function of impact force and the variation of the PDA‐SF sensors' colorimetric response as a function of force (F = 0–550 N) evaluated as the ratio (α) of the reflectance peak of the red phase (λ_red phase_ = 570 nm) with the blue phase (λ_blue phase_ = 651 nm). (c) Images of PDA‐SF paper sensors activated in the force range F = 0–880 N, corresponding fluorescence response (λ_excitation_ = 556 nm, λ_emission_ = 650 nm) as a function of force (F = 0–880 N).

The mechanochromic response of the PDA‐SF vesicles is assessed using paper‐based sensors that are activated with a drop dart testing setup (dart mass = 450 g; Figure 3a; Figure S8). Upon dart impact, the mechanochromic films exhibit a localized, visible color transition that is directly proportional to the force applied (Figure 3b). This color change is easily discernible to the naked eye and is confined to the area of the paper hit by the dart. The colorimetric response is linked to vesicle deformation, showing increased structural failure with higher energy impacts.

The PDA‐SF color transition can be quantified by acquiring reflectance data cubes using brightfield multispectral microscopy (Figure 3b; Figure S9), by measuring fluorescence intensity (Figure 3c), or by Raman spectroscopy (Figure S10). In brightfield analysis, the blue‐to‐red transition is visible within the impact force range of 𝐹 = 220–440 N. Lower forces do not produce a visible color change, whereas higher forces lead to partial damage to the paper substrate, evident from the increased white regions in the RGB micrographs of the dart impact zones. Multispectral imaging enables quantitative mapping of the impact force distribution on the PDA‐SF substrate, by providing pixel‐by‐pixel reflectance spectra in the acquired images. Figure 3b and Figure S11 show a false‐color mapping of the impact, showing that low forces (F1 = 220 N) cause only a small region of color change. Intermediate forces (F2 = 330 N) produce a full colorimetric shift across the impact area, showing an uneven color distribution, while higher forces (F3 = 440 N and F4 = 550 N) trigger an inward radial transition, with the strongest shift detected at the impact center. By analyzing the ratio between the PDA red phase reflectance intensity (I_red_ 570 nm, as extracted from liquid phase absorbance measurement) and its blue unperturbed phase (I_blue_ 651 nm), an increase in red phase intensity is observed in correlation with an increase in force, only to decrease for higher impact values, due to substrate damage (Figure 3b). For comparison, the mechanical response of PDA‐DMPC sensors under identical impact conditions is also evaluated (Figure S12); PDA‐DMPC show only a faint colorimetric shift visible to the naked eye, with minimal detectability in multispectral analysis, likely due to the poor alignment of PDA within the DMPC aggregates; These results highlight that using SF significantly improves the colorimetric response of the vesicles compared to DMPC‐based systems (Figure S13). Moreover, recent literature [62] has highlighted that confining mechanophores in vesicles or self‐assembled architectures can amplify color transitions by facilitating force transfer. Correlated Raman imaging with scanning electron microscopy (RISE) of PDA‐SF vesicles before and after activation shows a distinctive transition of the characteristic Raman peaks for the stretching of the C = C and C ≡C bonds. As expected [30, 74, 75], before activation, the PDA backbone is characterized by lower wavenumber vibrations (1454cm^−1^ and 2085cm^−1^, respectively), that shift to higher values upon activation (1511cm^−1^ and 2011cm^−1^, respectively).

Additional force transduction is provided by the changes in fluorescent properties of the mechanochromic sensor, given the presence of a fluorescent response in PDA's red phase (λ_Em_ = 650 nm), that is otherwise absent in the unperturbed blue phase (Figure S14) [76]. Analysis of the fluorescent emission of the mechanochromic paint expands its sensing range to F = 100 – 770 N in comparison to brightfield analysis (Figure 3c). Similarly to what was observed previously, for impact forces exceeding F = 700 N, the fluorescent response remains detectable but decreases due to partial damage to the paper substrate, which diminishes the signal. Other recent studies in the literature have proposed systems for the colorimetric detection of stresses in the kilopascal range [57, 58]. For comparison, considering that the contact area of our force sensor was a 1 mm diameter circle, the applied force range of 110–770 N corresponds to approximately 100–1000 MPa, thereby enabling the visualization of high‐impact stresses. These contrasting design strategies highlight that distinct architectures are required to cover different mechanical regimes: cascading devices are effective for weak stresses [58], whereas our protein‐stabilized PDA vesicles are tailored for high‐impact visualization.

**FIGURE 4:**
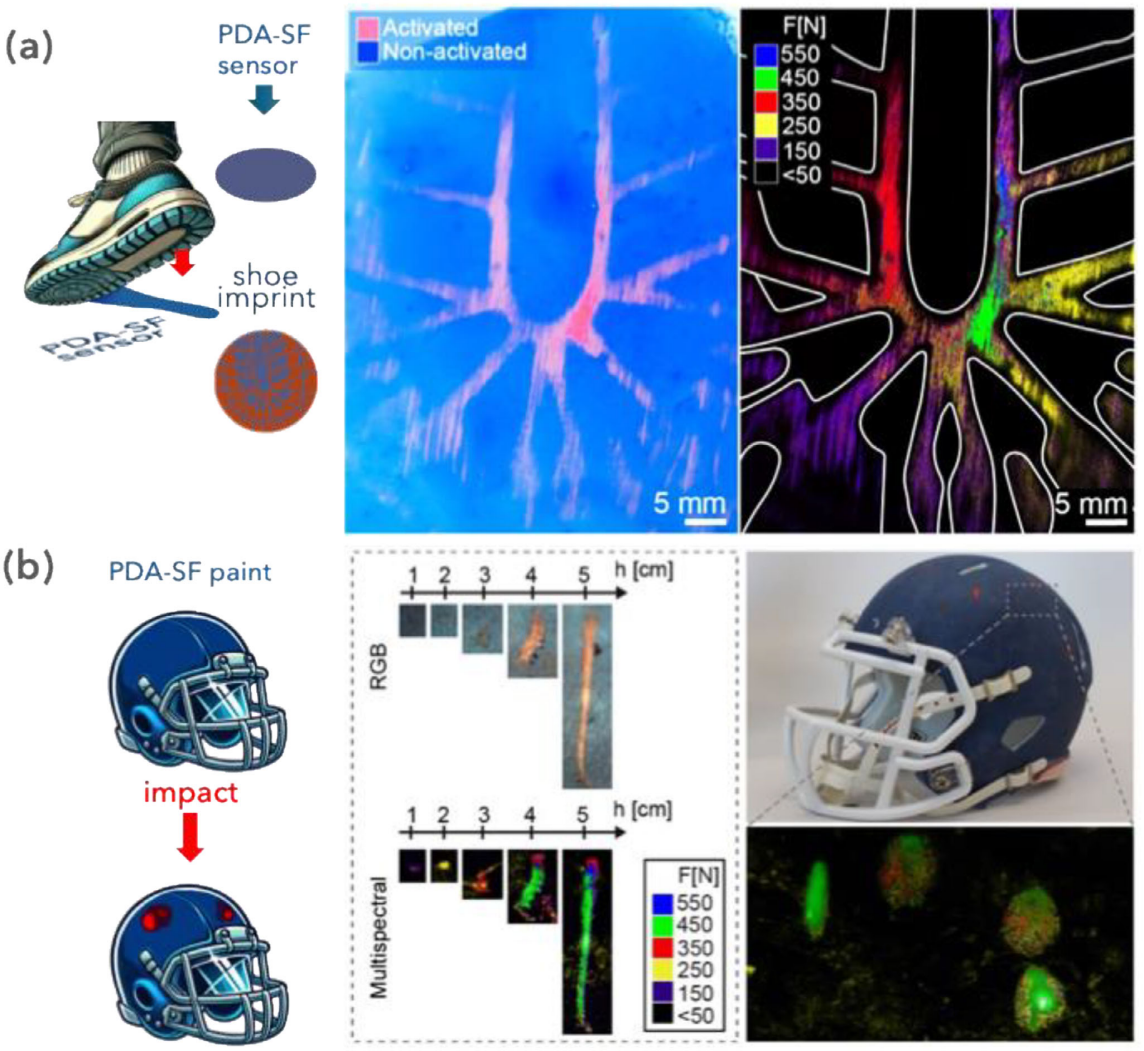
Applications of PDA‐SF paints on different surfaces (a) Schematic representation of the blue‐to‐red color transition of a mechanochromic PDA‐SF film applied on a polystyrene substrate upon stomping. Shown are the film image (left) and the corresponding false‐color composite calibrated multispectral image showing the color transition corresponding to the region of impact of the sole of the shoe and the distribution of the forces applied during stomping (right). (b) Application of PDA‐SF paints on a helmet. Calibration images in bright field reflection (top left) and corresponding false color multispectral images (bottom left) for a steel dart falling on the helmet from the height range 1–5 cm. Macroscopic picture of the helmet after impact of a steel cylinder on the helmet (top right) and corresponding false‐color composite multispectral image of the helmet showing the color transition in correspondence with the region of impact (bottom right).

**FIGURE 5:**
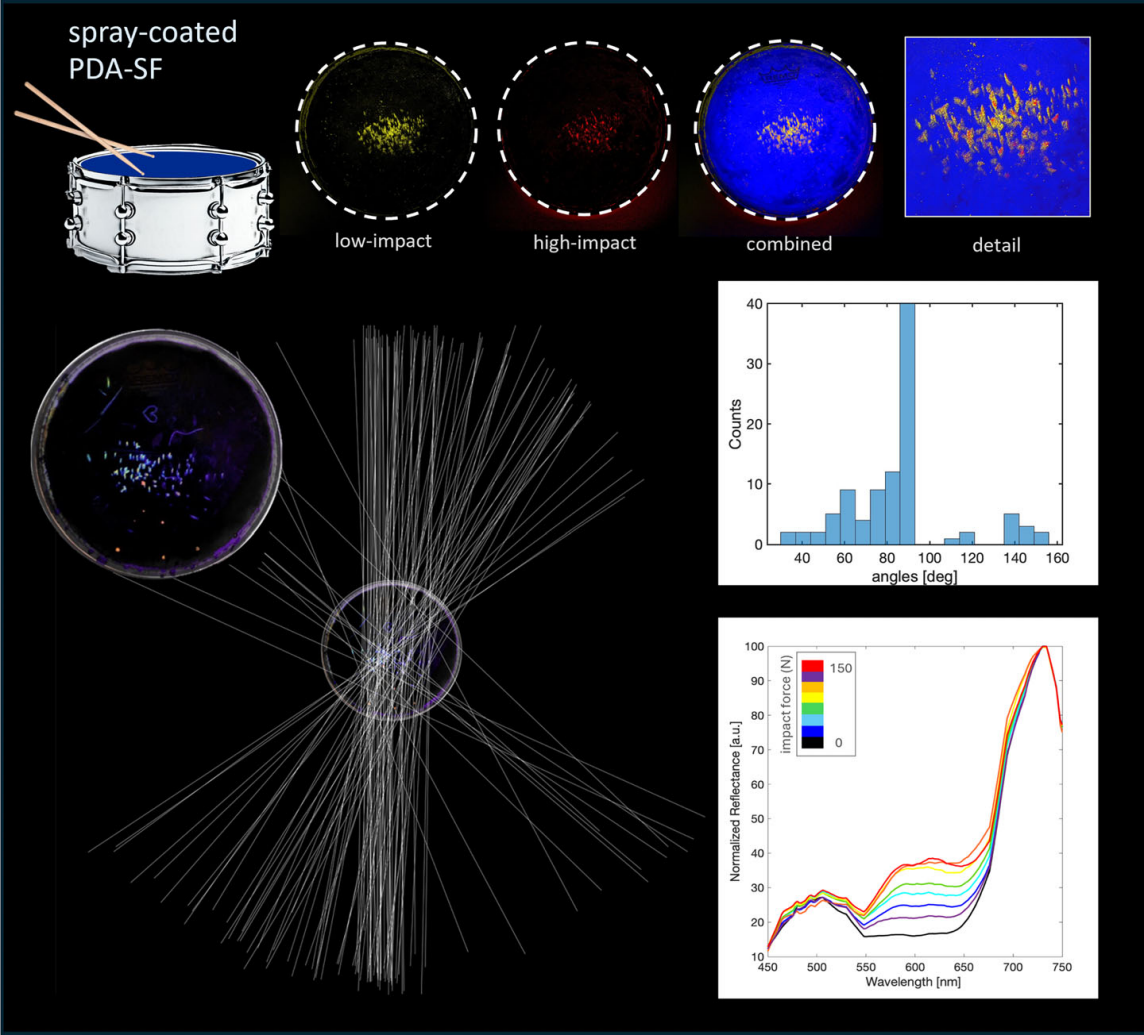
Analysis of force distribution on drum skin—PDA‐SF mechanochromic paint sprayed onto a drum skin mounted on a drumkit tom. The skin changes color during the song, tracing the musical performance of the artist. Typical forces range between <50 N and 200 N, and multispectral imaging is performed to cover this spectral range. The images show reconstructed multispectral analysis from the drum skin showing the distribution of low force hits, and high impact hits (either due to a single strong strike or due to accumulation of multiple weaker strikes on the same spot), and a combined image showing the two. The centroid of each hit is used to define the stick's direction upon impact onto the skin and represents them as a graphic rendition where 0° corresponds to the horizontal direction. Also shown are the statistics showing the distribution of stick direction and the spectral responses from data acquired with multispectral analysis of the drum skin. [Correction added on 20 January 2026 after online publication: figure 5 is updated.]

To demonstrate the versatility of this approach, representative prototype devices were fabricated by applying the PDA‐SF suspension onto a variety of surfaces of practical utility (Figures 4 and 5). The applicability of PDA‐SF vesicles as force‐monitoring for human gait is demonstrated by casting a mechanochromic vesicle layer on a polystyrene sheet and applying a stomp. This action induces a visible PDA transition, replicating the outsole's pattern in detail. The uneven force distribution expressed during the step is quantified by analyzing the blue‐to‐red transition on the sheet with multispectral imaging and associated calibration curves (Figure 4a; Figures S15 and S16). This approach enables clear visualization of the outsole regions that exert/experience the highest impact forces (F = 550 N), providing a detailed force distribution map across its surface.

A second demonstrator device is fabricated by painting a miniature of a football helmet replica with the sensors (Figure 4b). The helmet is subjected to drop dart testing in a similar manner to the paper‐based sensors. Multispectral analysis (Figure 4b, left) once again highlights how the forces are distributed across the helmet during impact. Specifically, when the dart is dropped from heights of 4 cm and 5 cm and slides along the helmet's curved surface, the force map clearly differentiates between the initial impact site and the slide‐off zone.

Within the primary impact site, closer inspection reveals distinct inner and outer areas that correspond to the dart's pointed geometry. Reflectance spectra from these tests also analyzed the forces that the helmet withstands during a series of hammer impacts (Figure 4b, right, Video S1). In this case, forces appear more uniformly distributed due to the hammer's flat surface, and image analysis helps differentiate impact dynamics, whether flat or cutting (Figure S17), or the geometry of the impacting object (Video S2). The force map revealed values around 250–450 N, consistent with typical hammering forces. Unlike planar PDA architectures, where sensitivity strongly depends on the direction of the applied stimulus [31], the 3D organization presented here—with randomly oriented PDA sheets—broadens the sensing range and makes the response less dependent on directionality.

Figure 5 shows an example in which silk‐PDA paint was applied onto drum skins. The as‐prepared skins are mounted on a drum kit, which is then used to perform a song. The drum skin changes color, tracing the musical performance of the artist, the distribution of force exerted by the drumsticks on the skin, and the location and distribution of the percussive impacts throughout the performance. This sort of “music analytics,” besides proving the versatility of the impact sensors, also opens a new approach in musical education and visualization. Analysis based on false‐color multispectral images allows tracing the direction of the sticks during the performance and allows correlating it with the drumstick impact. This visualization allows access to intangible aspects such as teaching “feel,” the skill that requires modulating force and angle of strike during playing in order to modulate tone, and obtain different sound outputs that are matched to the dynamics required by the tune. The multispectral false‐color maps in Figures 4 and 5 further reveal force‐distribution patterns that reflect the geometry and dynamics of each impact event, such as the characteristic outsole pressure zones during stomping, the directional loading imparted by the dart as it travels across the helmet surface, and the localized strike signatures on the drum skin. These results indicate that the spectral calibration established under controlled, fixed‐area impacts (Figure 3) can be extended to complex, real‐world contact scenarios, enabling quantitative visualization of force distributions even when the underlying contact area is irregular, deformable, or not directly measurable.

This study introduces a simple and scalable route to core–shell polydiacetylene–silk fibroin (PDA–SF) vesicles, marking the first demonstration of a PDA‐based mechanochromic sensor operating through this reinforced architecture. The synergistic integration of PDA with a semicrystalline silk shell produces a mechanically robust assembly that remains unresponsive under low and moderate loading yet undergoes a distinct chromatic transition when subjected to high‐impact stresses. As a result, PDA–SF coatings exhibit a quantitative activation window extending up to 770 N—substantially broader and higher than previously reported for colorimetric PDA systems.

A second distinguishing feature of this platform is the ability to extract quantitative, spatially resolved force‐distribution maps through multispectral analysis. By transferring spectral signatures calibrated under controlled impacts to irregular and dynamic real‐world conditions, the PDA–SF system enables direct visualization of how forces propagate across complex surfaces. This capability arises from the inherent mechanical stability of the core–shell assembly, which maintains structural integrity under impact and provides a reliable, interpretable chromatic response. The platform has broad utility, holding significant potential in human health and personal safety applications. For example, real‐time assessment of impact forces on sports helmets could enable early detection of concussions or traumatic brain injuries, while smart body armor or protective gear could signal dangerously high impacts before more severe damage or injury occurs. A further clinical advantage of these impact memory sensors is their ability to track and document the number and intensity of impacts, along with head acceleration, over time. [77] By maintaining a detailed “hit count,” these sensors enable comprehensive monitoring across practices, games, or even an athlete's entire career. Such long‐term tracking provides crucial data for assessing cumulative head impact exposure and can potentially inform injury prevention and management strategies.

Beyond human protection, additional applications could include structural health monitoring in aerospace, automotive, and civil infrastructure, and various other wireless force visualization applications, as exemplified through the drum skins. The improved uniformity of deposition and ease of fabrication further underscore the adaptability of PDA–SF vesicles, providing a cost‐effective, scalable platform for embedding robust sensing layers into a wide range of materials and devices.

## 3 Experimental Section

### 3.1 Materials

A total of 10–12 pentacosadyenoic acid, sodium carbonate, lithium bromide, and chloroform were purchased from Sigma‐Aldrich and used without any further purification. 1,2‐dimyristoyl‐sn‐glycero‐3‐phosphorylcholine (DMPC) is purchased from AVANTI Polar Lipids and is used without any further purification. Bombyx mori cocoons were purchased from Tajima Shoji (Japan).

### 3.2 Silk Fibroin Solution

The silk solution is prepared following an established protocol [78] as follows: *Bombyx mori* cocoons were degummed by cutting and boiling 10 g of cocoons in 4 L of 0.8 m Na_2_CO_3_ solution for 30 min to remove sericin. The washed and dried fibers were then dissolved in 9.3 m lithium bromide solution for 3 h at 60°C. The obtained silk solution is dialyzed against Milli‐Q water using a 3.5 MWCO nitrocellulose dialysis tubing for 3 days to remove residual lithium bromide. The silk solution is then filtered with nitrocellulose filter (70 µm, Falcon cell strainers), centrifuged (Beckman Coulter Allegra X‐14 Centrifuge, rotor FX6100) at 10200 rpm for 20 min at 4°C, and then filtered again to remove debris. The silk solution concentration is determined gravimetrically. Reconstituted silk fibroin solution is concentrated, as needed, up to 5% by pouring it into a 3.5 MWCO dialysis tubing and placing it into a drying chamber. Finally, the solution is stored at 4°C until further use.

### 3.3 Synthesis of PDA‐Based Vesicles

The general protocol [70] for the preparation of PDA liposomes involves first the dissolution of the DA monomers, along with DMPC, in chloroform to create an even distribution of monomers. Chloroform is then evaporated by a N_2_ stream, leaving a white film behind. Since SF is completely insoluble in chloroform, for the synthesis of the PDA‐SF vesicles, the silk solution is added to a dried DA film after chloroform evaporation. Briefly, 10 mg of PCDA were dissolved in 15 mL of chloroform. The solvent is then evaporated, and either 10 mg of DMPC or 100 mg of silk was added for the synthesis of either PCDA‐DMPC or PCDA‐SF, respectively.

The resulting mixture is resuspended in MilliQ water and dispersed by sonication at T ≥ 80°C, the phase transition temperature (Tm) of PCDA [79]. The resulting solution is then filtered through a nylon membrane (40 µm, Falcon cell strainers) to remove any aggregates. The solutions were tip‐sonicated (Misonix Sonicator model XL‐2000) at a power of 240 W for 15 min. To promote liposomes' stability and the alignment of the DA backbone, the colloidal suspension is stored at 4°C overnight. The solution is then centrifuged using a Beckman Coulter Allegra X‐14 Centrifuge equipped with a swinging bucket rotor (SX4750A) at 1000 rpm for 10 min to remove the bigger vesicles and aggregates; the supernatant is then collected and further centrifuged three times at 4300 rpm for 3 h to concentrate the vesicles. After this, the pellet is collected and used as a starting batch for the fabrication of the mechanochromic sensors. The concentration of the PDA‐SF solution is measured gravimetrically with C_PDA‐SF_ = 7 mg mL^−1^ and C_PDA‐DMPC_ = 0.5 mg mL^−1^.

The developed synthesis process is also successfully scaled up; starting from 5 g of silk and 500 mg of PCDA in a 500 mL batch gives ∼ 3.96 g of PDA‐SF vesicle suspension with a yield of ∼ 72%.

### 3.4 FTIR

Fourier‐Transform Infrared Spectroscopy (FTIR) spectra of SF‐PDA vesicles were acquired using a Bruker Invenio S Diamond ATR with a resolution of 1 cm^−1^ within a scan range between 400 cm^−1^ and 400 cm^−1^ and with the accumulation of 64 scans. FTIR spectra of SF were obtained from dried films of 5% solution of SF, since SF without PCDA does not form vesicular structures (Figure S4) nor precipitates during centrifugation.

### 3.5 UV–Vis Spectroscopy

UV–vis spectra of 1 mg mL^−1^ PDA‐SF or PDA‐DMPC vesicle suspension were acquired using a Biotek synergy HT plate reader. The liquid suspension of PDA sensors was activated by exposing them to the light of a VL‐215.G 60 W UV lamp for 2 min. The energy delivered by the lamp is 2.5 mW cm^−2^ measured with S302C—Power Sensor Head, Surface Absorber (Thorlabs), and the relative UV dose (mJ cm^−2^) can be quantified by multiplying the power (mW) by the exposure time (s).

### 3.6 Zeta Potential

To determine the zeta potential of PDA‐SF and PDA‐DMPC, 1 mg·mL^−1^ suspensions were prepared in deionized (DI) water and measured using a NanoBrook ZetaPALS (Brookhaven Instruments, Holtsville, NY). The zeta potential values were determined using the Smoluchowski model.

### 3.7 Paper‐Based Sensor Preparation and Drop Dart Testing

Paper‐based sensors were obtained by laser cutting (Trotec Speedy 300 laser cutter) 120 lb cardstock paper 300gsm (Desktop Publishing Supplies) in 2.5 cm squares and by drop casting 50 µL of 2 mg mL^−1^ either PDA‐SF or PDA‐DMPC suspension on them. The sensors were dried at room temperature and activated by UV irradiation for 2 min (UVP XX‐Series UV Bench Lamp, 115 V).

The sensors’ mechanical activation is performed by using a custom‐made drop tower mechanism designed for impact testing (Figure S8). A 150 × 25 mm custom‐made steel dart with a diameter of 1 cm is positioned on a pullable pin set at an adjustable height between 1 cm and 6 cm above the sensor. When the pin is pulled, the dart falls and hits the material, thus activating the colorimetric transition in the region directly hit by the dart. For the calculation of the impact energy, the potential energy from the falling object (the dart) is assumed to convert to kinetic energy without dissipation [80, 81, 82] so that the 450.0 g dart falling from 1 cm height causes 110 N of mechanical impact, considering a deformation of d = 0.3 mm; each increase of falling height of 1 cm induces an increase in the delivered force of additional 110 N (Table S3). Velocity, energy, and impact force from different falling heights are summarized in Table S3.

For consistency with the literature, we also report stresses calculated by normalizing the measured forces to the nominal contact area of the dart tip (1 mm diameter circle). This yields impact forces in 100–800 N as stresses in the 100–1000 MPa range.

### 3.8 Mechanochromic Polystyrene Sheet

The polystyrene hexagonal substrates (side l = 50 mm) were obtained from weighing boats (Fisherbrand Polystyrene Antistatic Weighing Dishes) and exposed to UV light at λ = 254 nm for 3 h to increase the surface's wettability. Then, the PDA‐SF suspension is cast on top, forming a film with a density of 2 mg cm^−2^ which is then activated with UV light for a further 30 s to polymerize the vesicles.

### 3.9 Mechanochromic Helmet

A bike helmet is brushed with 50 mL of PDA‐SF vesicle suspension with a concentration of 10 mg/mL mixed with white acrylic. After drying, the coating has been activated with 30s exposure to UV light.

### 3.10 Optical Microscopy

A customized Olympus Inverted IX71 microscope equipped with a DSLR (digital single‐lens reflex) camera (Canon Rebel T1i) and a halogen lamp (Olympus, U‐LH100L‐3) as a light source is used to perform optical microscopy. Bright‐field reflection images were collected using a 4× (Olympus, UPlanFL N, numerical aperture 0.13) objective. To quantify the reflectance of the paper‐based sensors, the microscope is coupled to a multispectral camera (CRI, Nuance EX). The paper‐based sensors were observed in bright‐field reflection, and the spectral cubes were acquired in the spectral range λ = 450–750 nm with a step size of 3 nm. The software Nuance 3.0.2 is used to acquire and unmix the spectral cubes into individual spectral components. When the activation of the PDA‐SF sensors induced deformation in the paper substrates, multiple micrographs were acquired at different focal points and combined using the focus‐stacking algorithm using the software ImageJ. The optical image reported in Figure 2a was acquired using a Zeiss LSM 900 Imager.Z2m microscope in bright field reflection equipped with a LED lamp, a C‐Epiplan‐Apochromat 50×0.95 NA DIC objective, and a Zeiss Axiocam 503 color camera. The image was obtained by acquiring a z‐stack over 3.28 µm (9 slices) and processing it through extended depth of focus using the Zen Blue software.

### 3.11 Colorimetric Analysis

For the UV activation, the colorimetric analysis of the paper‐based sensors is performed, evaluating the ratio I_red_/I_blue_ with I_red_ being the reflectance intensity at λ_1_ = 570 nm and I_blue_ the reflectance intensity at λ_2_ = 651 nm. The spectral positions for λ_1_ and λ_2_ were extrapolated from the absorbance spectra of the liquid SF‐PDA solution: λ_1_ = 570 nm corresponds to the maximum absorbance peak for the red phase of the PCDA, while λ_2_ = 651 nm to the blue phase. The reflectance spectra were acquired using the multispectral camera for three different replicates of the sensors; the measurements were performed in triplicate and reported as mean values (Mean) ± standard deviation (SD).

### 3.12 Fluorescent Analysis

Triplicates of paper‐based sensors were subjected to different impacts using drop dart testing. The sensors were cut into 4 mm circles, and the fluorescence (endpoint fluorescence measurement) was quantified using the BIOTEK plate reader Synergy HTX. The red phase of the sensor exhibits fluorescence, while the blue phase does not. The emission is measured at 660 nm with excitation at 556 nm.

### 3.13 Electron Microscopy

The PDA‐DMPC and PDA‐SF vesicle solutions were cast on a thin cover slip, dried at room temperature, and mounted on aluminum stubs using conductive carbon tape. To ensure electrical conductivity, the stubs were sputtered with ∼10 nm of gold using an Emitech SC7620 sputter coater. The prepared specimens were imaged in top view using a Zeiss EVO MA10 SEM with a secondary electron detector at 10 kV and 7 to 9 mm as the working distance. The SEM images were analyzed with the software ImageJ to determine the diameter of the PDA‐SF vesicles: the values were reported as mean values ± standard deviation (SD) for N = 189 vesicles. To image the internal morphology of the PDA‐SF vesicles, the following protocol [83] is used: the PDA‐SF ink is cast on a glass coverslip previously covered with a layer of transparent polish (nitrocellulose in butyl acetate). A second layer of polish is applied to ensure complete embedding of the particles. The particles were then placed in an Argon atmosphere, cooled in liquid nitrogen, and mechanically cryo‐fractured. The fragments containing the particles were then mounted on aluminum stubs using conductive carbon tape and sputtered with ∼10 nm of gold.

### 3.14 Elemental Analysis

The PDA‐SF vesicle solution is cast on a thin cover slip, dried at room temperature, sonicated for 2 min in liquid nitrogen, and mounted on aluminum stubs using conductive carbon tape. To ensure electrical conductivity, the stubs were coated with ∼10 nm of chromium using a Quorum Q150T ES Plus sputter coater. The specimen is imaged using a Zeiss Sigma 300 Field Emission Scanning Electron Microscope (FE‐SEM) with an InLens electron detector at 5 kV with 8.8 mm as the working distance. An Oxford Instrument UltimMax 65 EDX detector with the Atzec 6.0 software is used to assess the elemental composition of the sample.

### 3.15 Raman Spectroscopy

The conformational change of the PDA‐SF vesicles upon activation was measured using a Zeiss Sigma 300 Field Emission Microscope (FE‐SEM) with an in situ confocal Raman setup (RISE) [84]. Samples were mounted on aluminum stubs using conductive carbon tape, imaged as is with no additional coating (EHT = 0.8 kV, with a secondary InLens detector, and working distances set to WD = 4.3 mm), and subsequently moved under the Raman detector.

Raman spectra were acquired using a Witec WRL‐532‐E‐100‐TP laser (λ_excitation_ = 532 nm, p = 75 mW) equipped with a UHTS300VIS imaging spectrograph (600 g/mm grating), a PE cooled CCD, and a 3D piezoscan stage (250 µm × 250 µm × 250 µm). The laser was focused through a Zeiss LD EC Epiplan‐Neofluar Vac Dic 100x objective (WD = 4 mm, NA = 0.75). Spectra were acquired at laser power p = 0.1 mW, integration time t = 0.1s, accumulations N = 5, while working at room temperature. The laser was focused inside the vesicle to acquire the PDA signal with minimal interference from the silk shell. Acquired Raman spectra were further processed using the Witec Control 6.2 software to perform cosmic ray removal, background subtraction, and smoothing using the Savitzky‐Golay method.

### 3.16 Drum Skin Analysis

False‐color multispectral images were imported into a vector graphics editor software (Adobe Illustrator 2025) to determine the direction of the drumstick strikes. The centroid of each impact mark, along with the associated color distribution, was used to guide the placement of the vectors representing the strike direction. When no clear direction could be determined from the centroid, the corresponding centroid was omitted from the analysis. The angles of each vector with respect to the horizontal direction (θ = 0°) were measured and subsequently imported into the software Matlab R2024b for further analysis and visualization.

### 3.17 Images

Digital images of the paper‐based sensors and of the prototypes were taken with a DSLR (digital single‐lens reflex) camera (Canon 5D mkIII). The RAW image files were adjusted for exposure and contrast.

## Conflicts of Interest

The subject of this paper is being protected through patent filings by the technology licensing office of Tufts University.

## Supporting information




**Supporting File 1**: advs73572‐sup‐0001‐SuppMat.docx.

## Data Availability

The data that support the findings of this study are available from the corresponding author upon reasonable request.
