# Supplementary material for: Visualizing and Quantifying Impact with Mechanochromic Sensing Paints Based on Self‐Assembled Polydiacetylene‐Silk Core‐Shell Vesicles
Source: Adv Sci (Weinh). 2026 Jan 4;13(19):e18144. doi: 10.1002/advs.202518144 (PMC13045206; doi:10.1002/advs.202518144)
Supplement: Supplementary file 1 — Supporting File 1: advs73572‐sup‐0001‐SuppMat.docx. [file ADVS-13-e18144-s001.docx]

Supporting Information

Visualizing and quantifying impact with mechanochromic sensing paints based on self-assembled polydiacetylene-silk core-shell vesicles

Marco Lo Presti^1,#^, Giulia Guidetti^1,#^, Elisabetta Ruggeri^1^, Terri Lyne Carrington^2^, and Fiorenzo G. Omenetto^3,4,5,^*


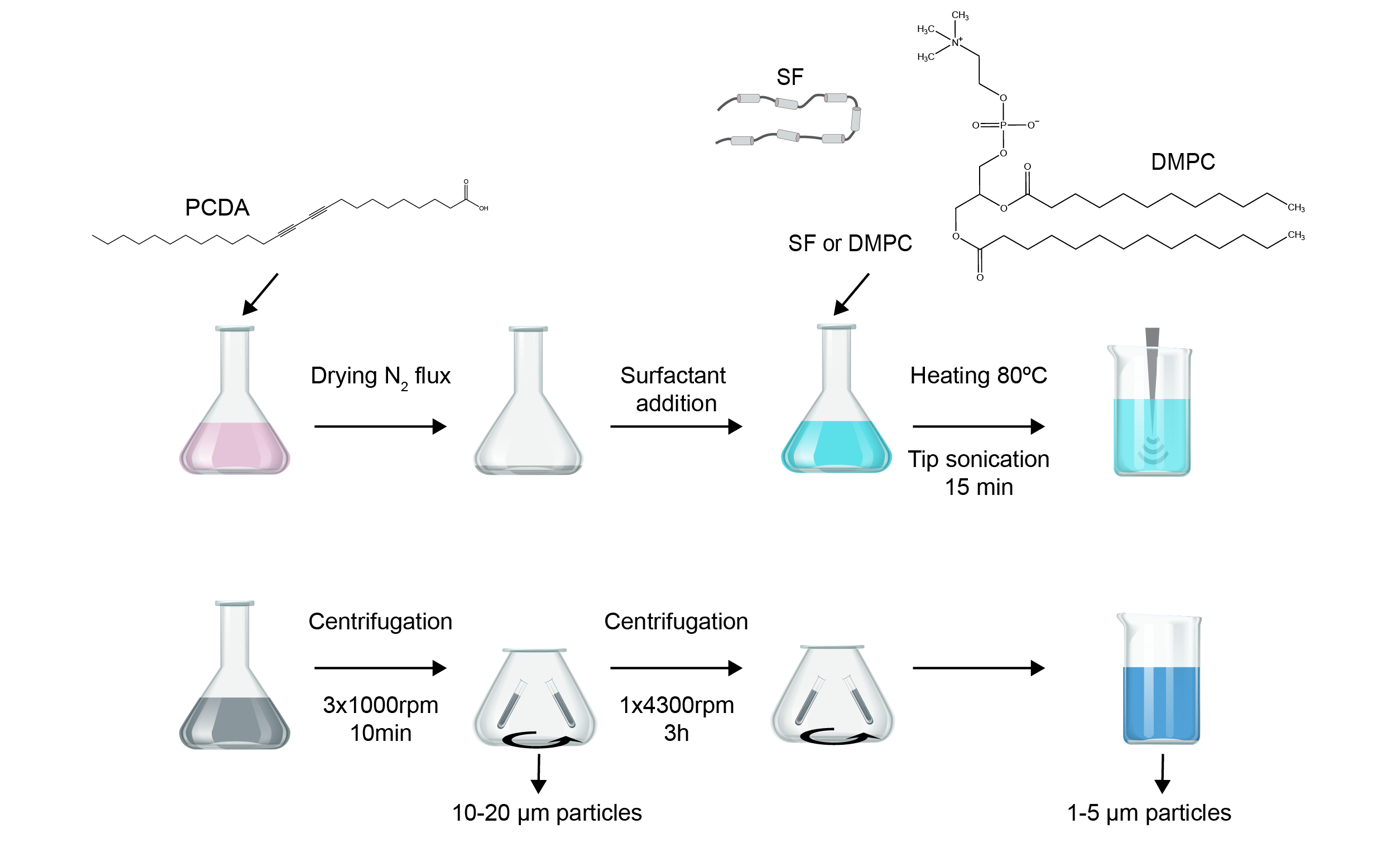


Figure S1. Schematics representation of the preparation protocol for PCDA-SF and PCDA-DMPC vesicles. PCDA is dissolved in chloroform and the solvent is evaporated through nitrogen flux. An aqueous solution of either SF or DMPC is added to the solid film of PCDA. The solution is stirred in a water bath at T=80°C and then tip sonicated for t=15 min. The suspension is then centrifuged thrice a 1000 rpm for 10 min to remove large vesicles (diameter >10μm) and aggregates, and a further centrifugation step at 4300 rpm for t= 3 h allows the recovery of the smaller vesicles (pellet) used to fabricate the mechanical sensors.


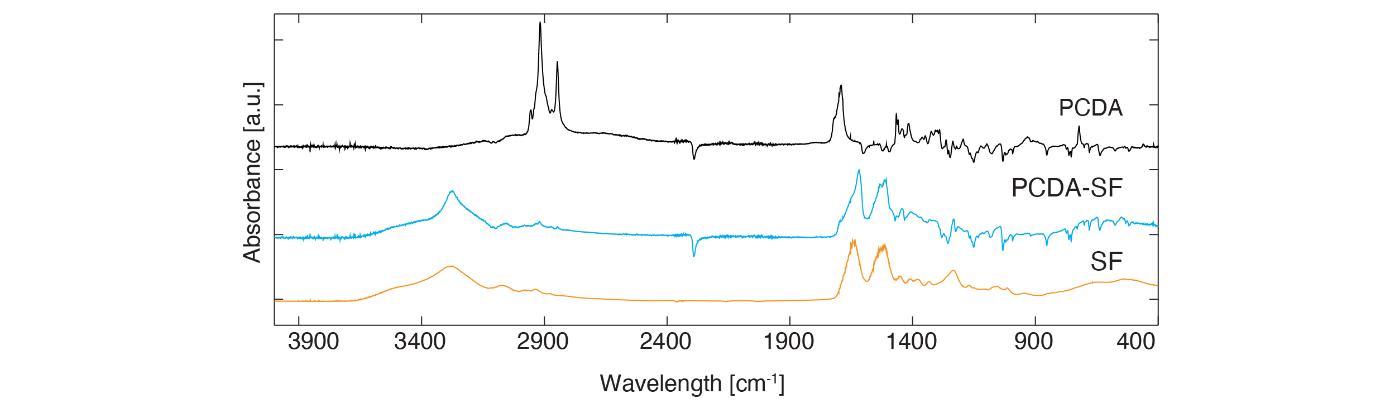


**Figure S2.**
FTIR peaks attribution:

The three main peaks of PCDA^86^ are visible at 2919 cm^-1^ (ν_a, CH2_), 2848 cm^-1^ (ν_s, CH_), and 1694 cm^-1^ (ν, _C=O_), while the three main bands of SF^87^ at 1700–1600 cm^–1^ (ν_C=O_ amide I), 1600–1500 cm^–1^ (ν_s, C-N_ amide II) and 1330–1230 cm^–1^ (δ_s, C-N_ amide III).


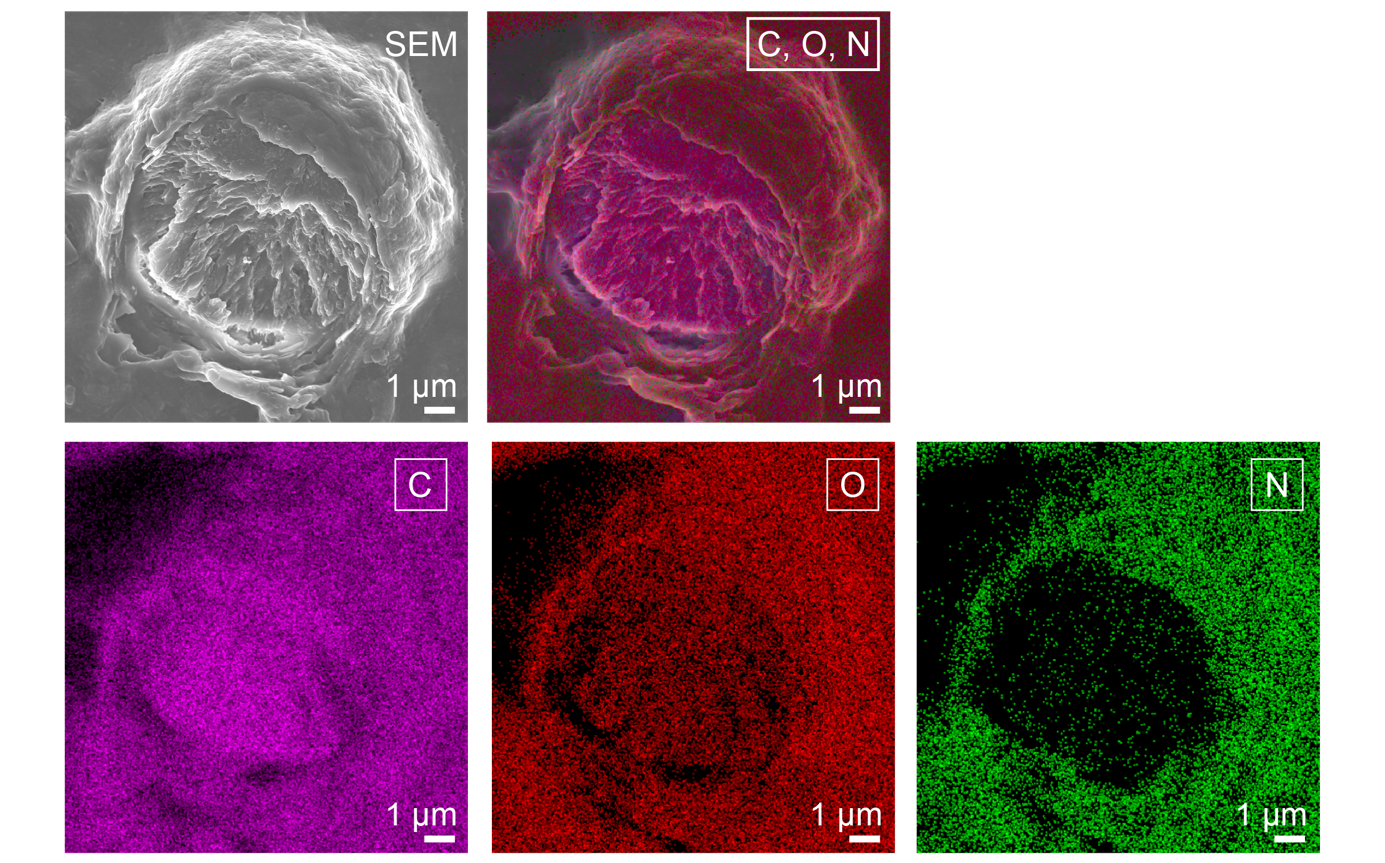


Figure S3. SEM Energy Dispersive X-ray Spectroscopy (SED-EDX) analysis of a core-shell PD-SF vesicle. SEM image (top left), and false color images of the detected elements combined (calcium, oxygen, and nitrogen). Mapping of individual elements is also reported for carbon (C, Kα1,2), oxygen (O, Kα1), and nitrogen (N, Kα1,2). Top left corner of the images show the shadow effect caused by the three-dimensionality of the sample.


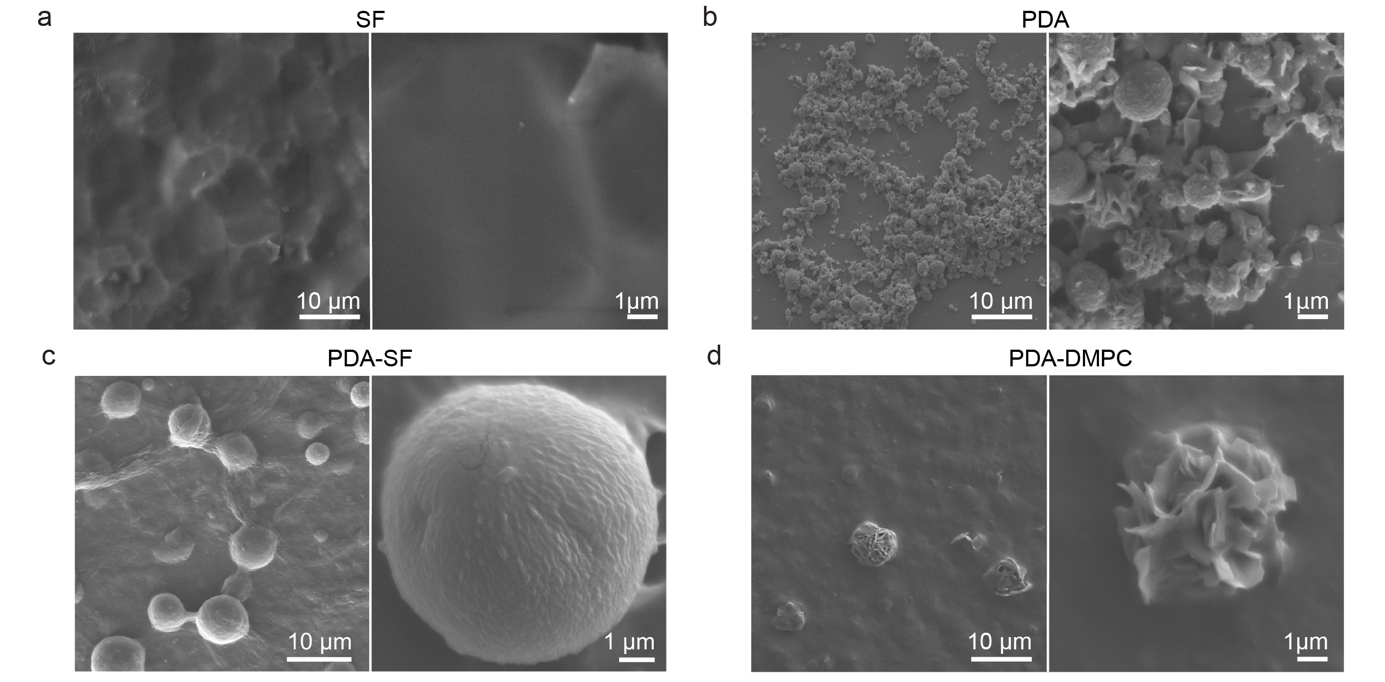


Figure S4. SEM top-view images at low (left) and high (right) magnification for (a.) SF only, (b.) PDA only, (c.) PDA-SF vesicles, and (d.) PDA-DMPC vesicles. No core-shell vesicles can be observed for SF only, while PDA only produces vesicles with heterogeneous surface morphology, and PDA-DMPC vesicles have exposed PDA sheets.


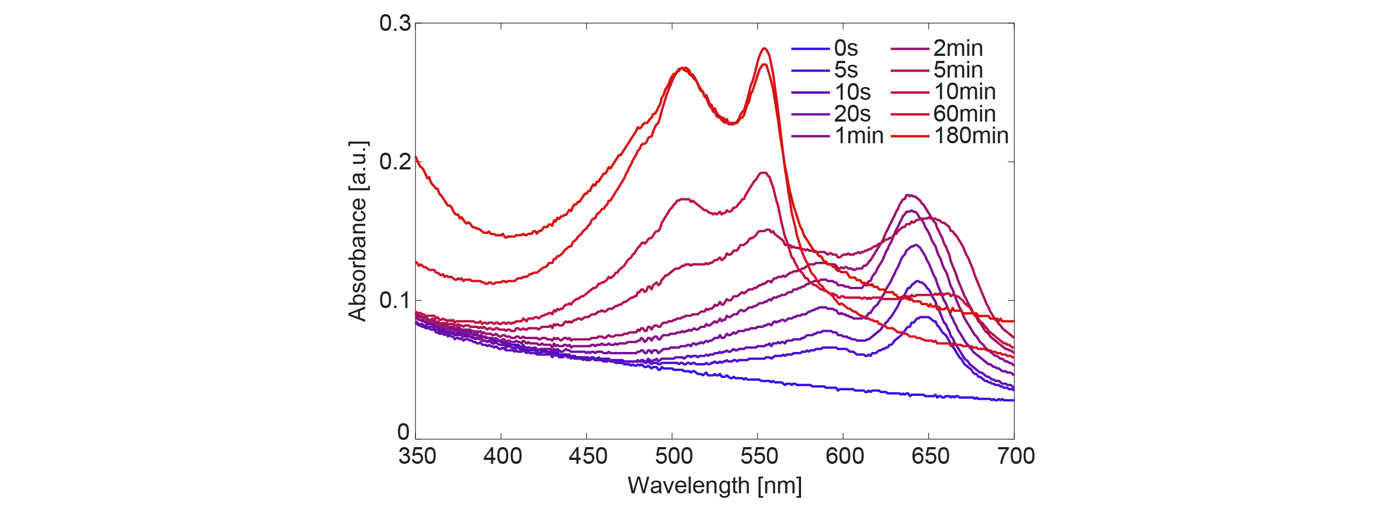


**Figure S5.** PDA-DMPC absorbance spectra as a function of wavelength after UV activation for 0 s-180 min.


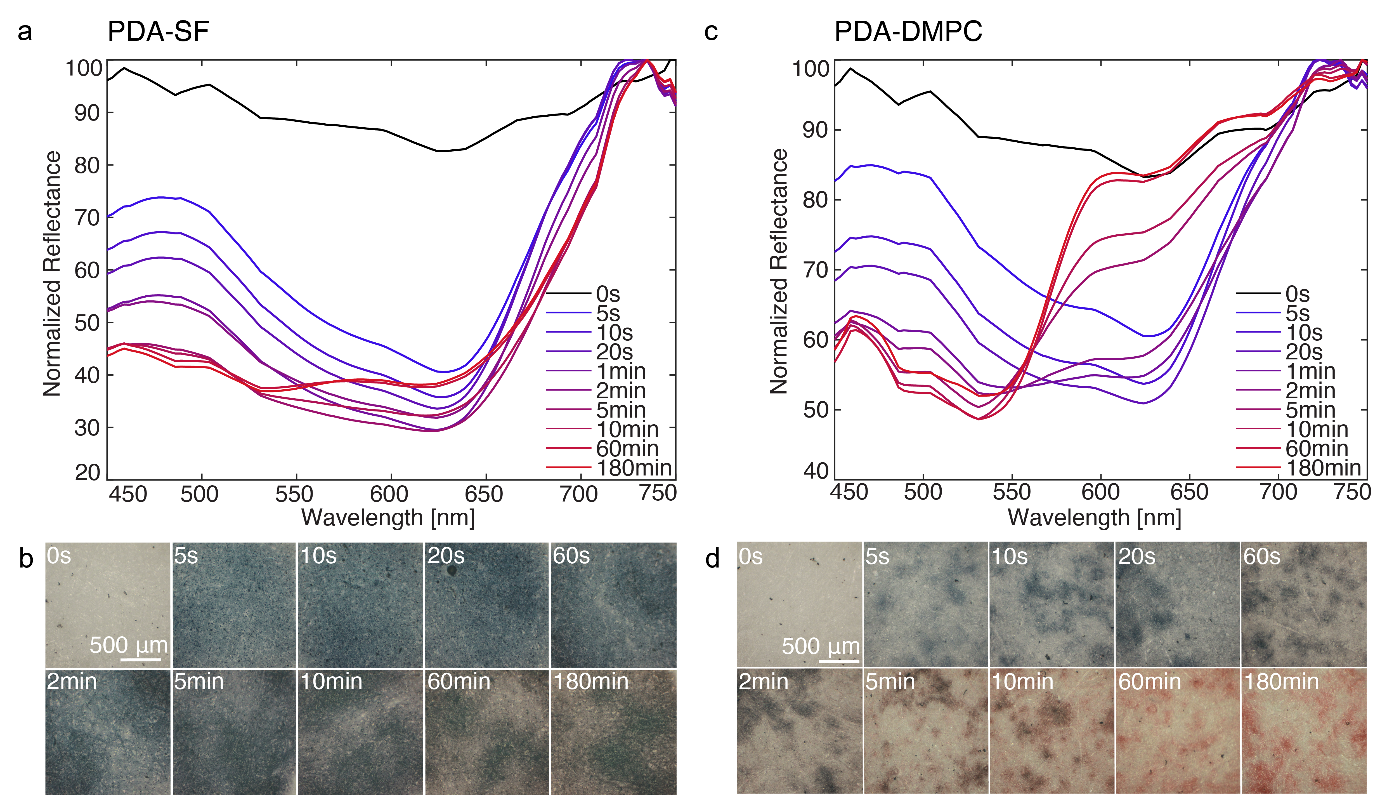


Figure S6. reflectance spectra as a function of wavelength of paper-based sensors after UV activation for 0 s -180 min of a) PDA-SF and b) PDA-DMPC and c,d their corresponding brightfield reflectance micrographs.


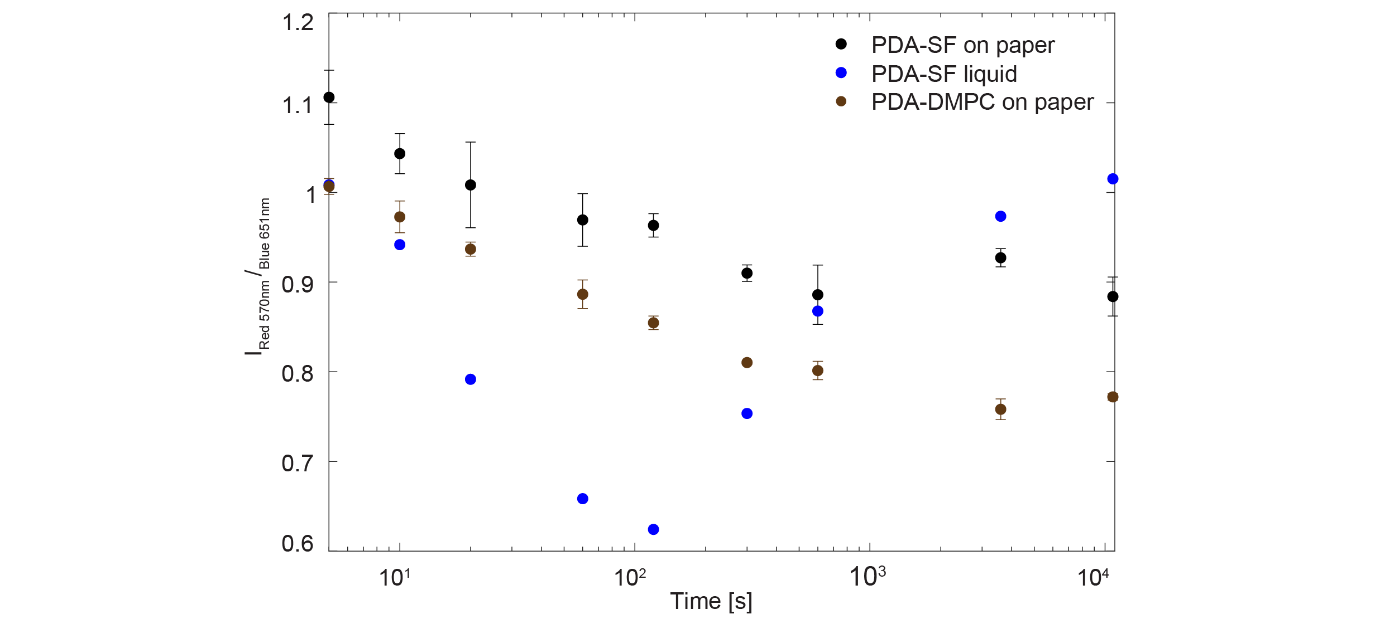


Figure S7. Variation of the PDA colorimetric response as a function of UV exposure time (0 s -180 min) quantified as the ratio of the peak of the red phase (λ_blue phase_ = 651 nm) with the red phase (λ_red phase_= 570 nm, as extracted from liquid phase absorbance measurement) for the PDA-SF solution, and for PDA-SF and PDA-DMPC paper-based sensors. For the solution, the peak intensity is extrapolated from the absorbance spectra, while for the paper-based sensors from the reflectance spectra.


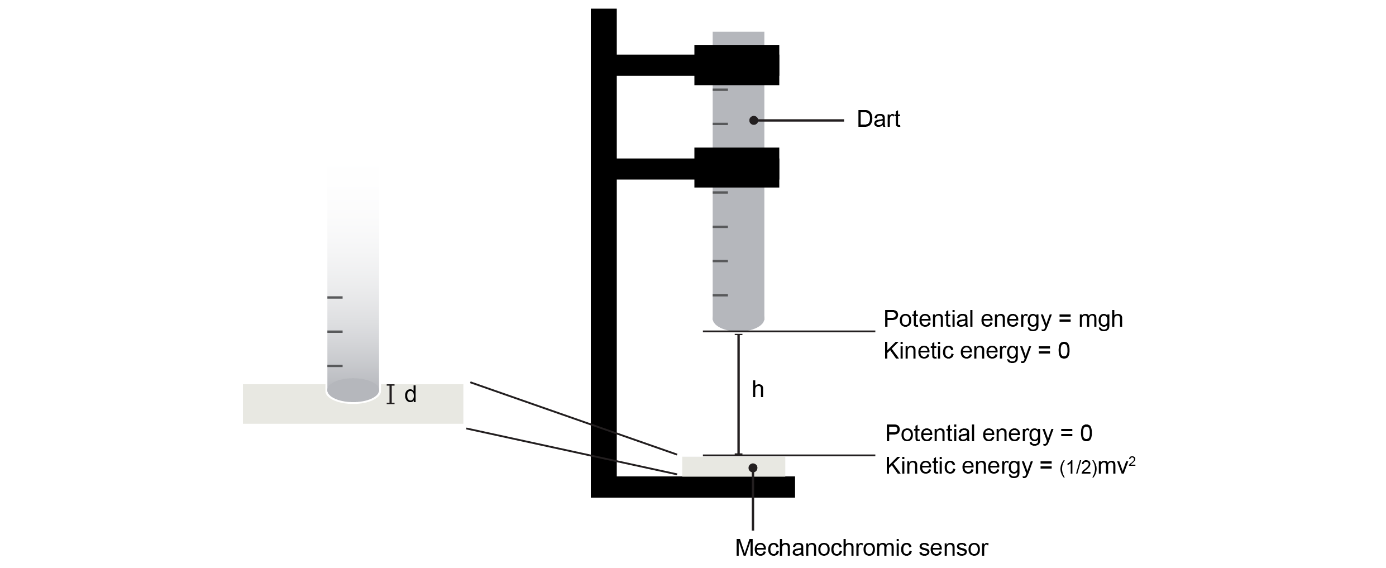


Figure S8. Schematic representation of the mechanical setup used to activate the mechanochromic paper-based sensors. The dropping height *h* is measured from the top of the sample to the bottom of the stainless-steel dart before dropping.


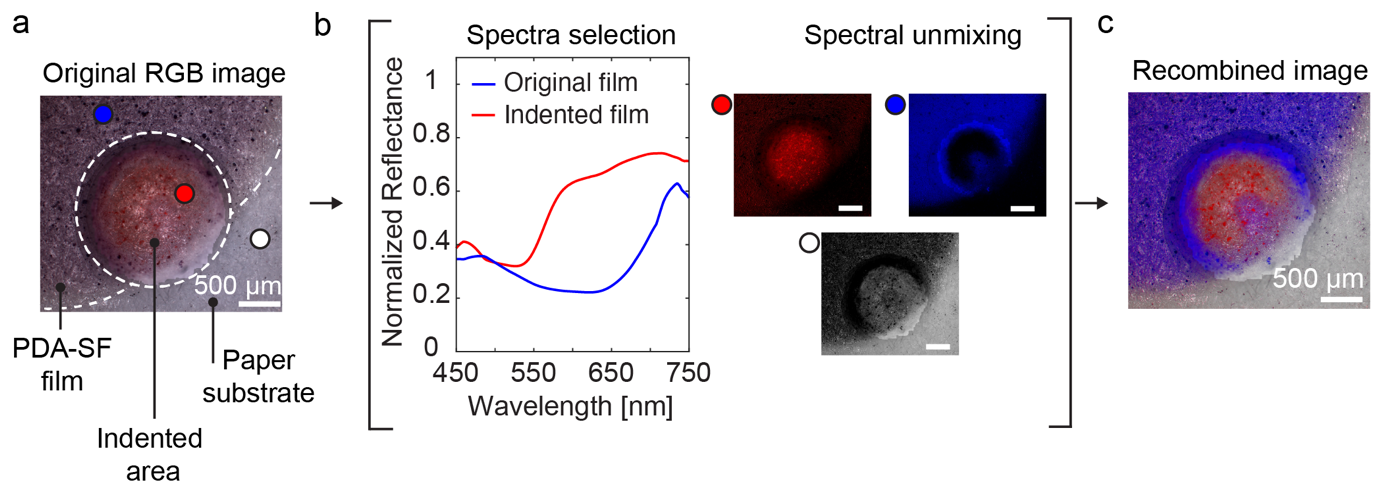


Figure S9. Multispectral analysis of PDA-SF mechanochromic sensors. a. The original RGB reflectance image of an indented PDA-SF sensor is used as a reference for the selection of the characteristic spectra of the system. b. For each sensor, spectral responses of the active region and of the background (paper) were selected: red and blue reflectance spectra as a function of wavelength were acquired from an indented area of the PDA-SF film and a non-indented area of the film, respectively. These spectra were used to unmix the reflectance response in individual spectral bands (represented by false-colors) and to c. obtain the reconstructed false-color composite image. Scale bar: 500μm.

Figure S10. Raman spectra of blue-phase and red-phase PDA-SF vesicles demonstrating the conformational transition upon activation. The characteristic Raman peaks of the double and triple bond stretching (C=C and C ≡C) in the blue phase (1454cm^-1^ and 2085cm^-1^, respectively) shift to higher wavenumbers (1511cm^-1^ and 2011cm^-1^, respectively).


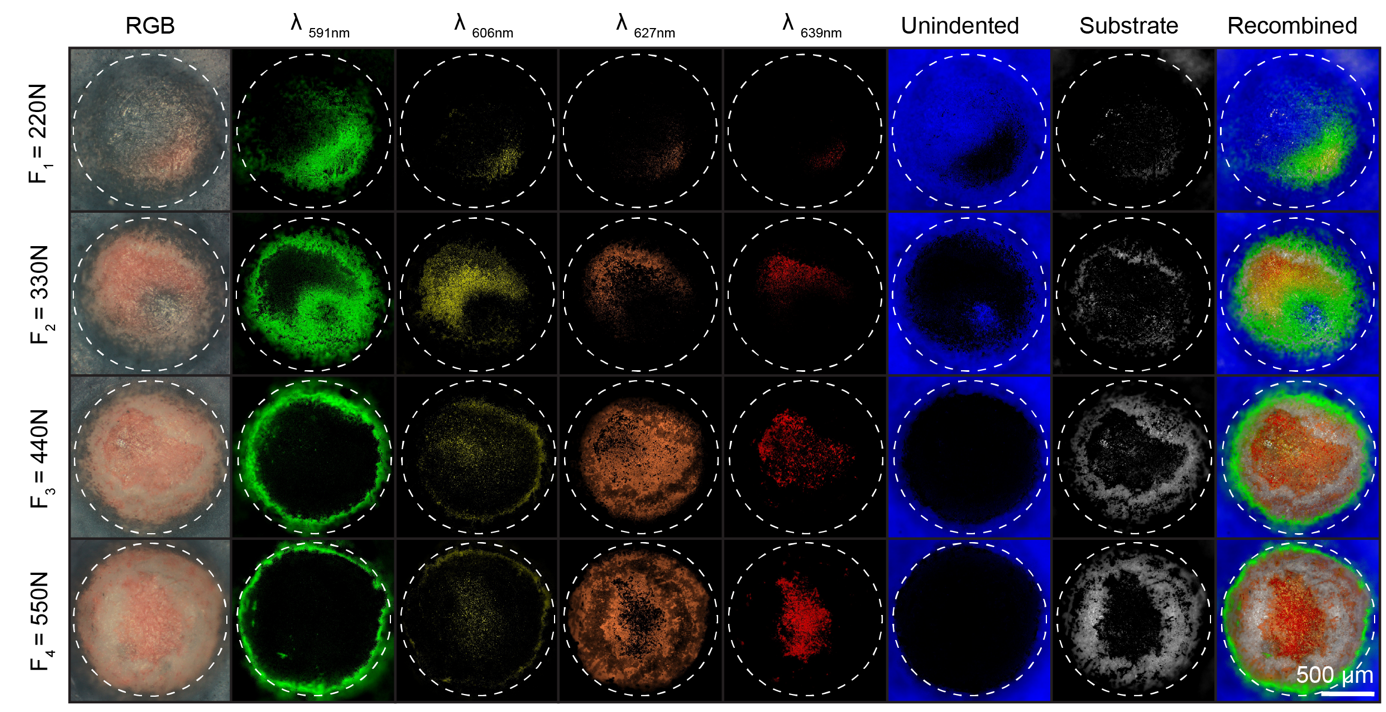


Figure S11. Quantitative colorimetric analysis of PDA-SF paper-based mechanochromic sensors. Multispectral analysis of PDA-SF sensors for the impact force range F = 220 – 550 N showing the original micrograph (RGB) and the false-color spectral bands corresponding to the characteristic reflectance spectra for impact for each impact force, for the non-indented region (Unindented), and the substrate (Substrate). The reconstructed image (Reconstructed) shows the impact distribution in the sensors.


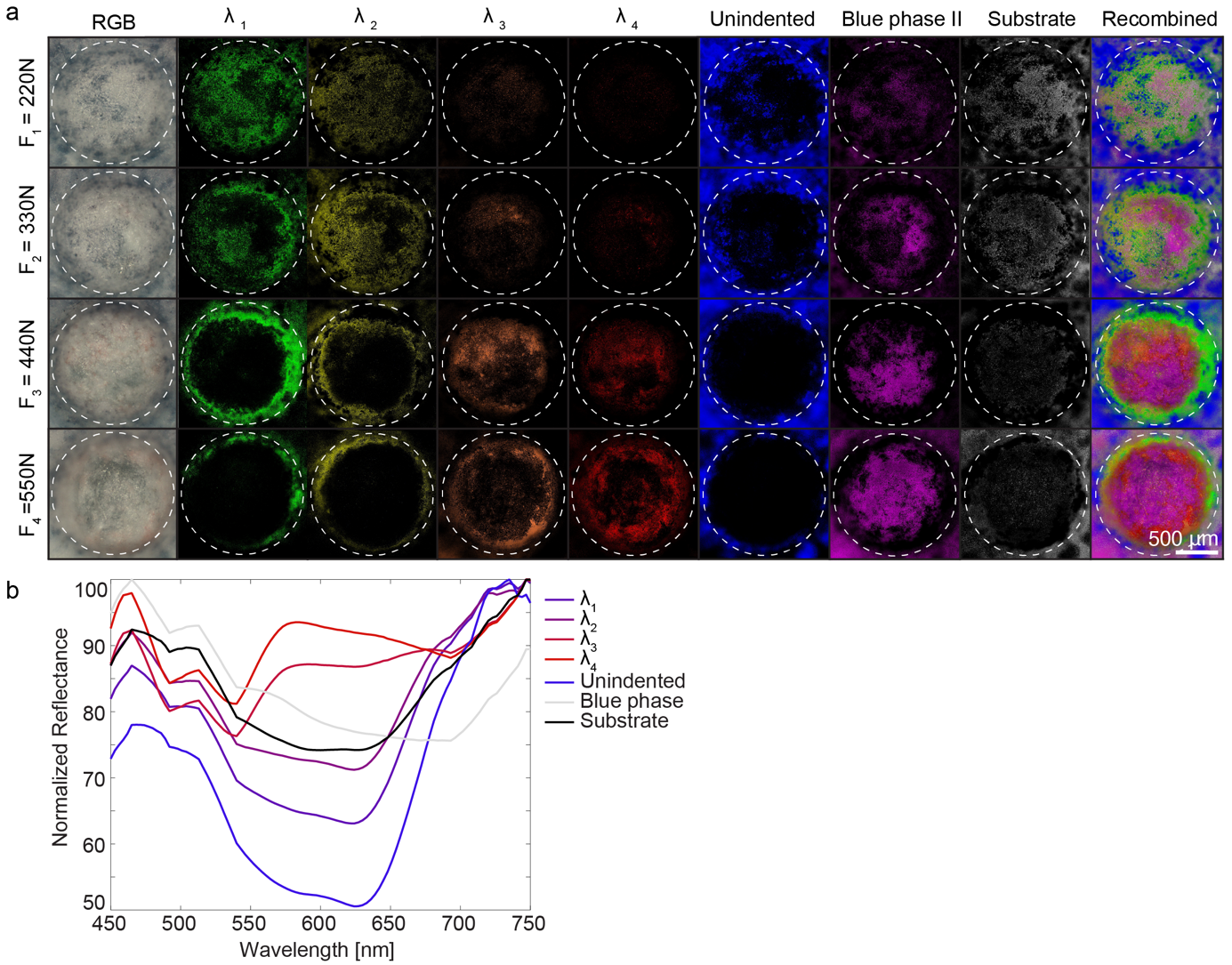


Figure S12. Quantitative colorimetric analysis of PDA-DMPC paper-based mechanochromic sensors. a. Multispectral analysis of PDA-DMPC sensors for impact force range F = 220 - 550 N showing the original micrograph (RGB) and the individual false-color spectral bands corresponding to the main colorimetric response for each investigate force, the additional blue phase observed in the PDA-DMPC (Blue phase II), the un-indented region (Unindented) and the substrate (Substrate). The reconstructed image (Reconstructed) exemplifies the impact distribution in the sensor. b. Corresponding normalized reflectance spectra for PDA-DMPC sensors.


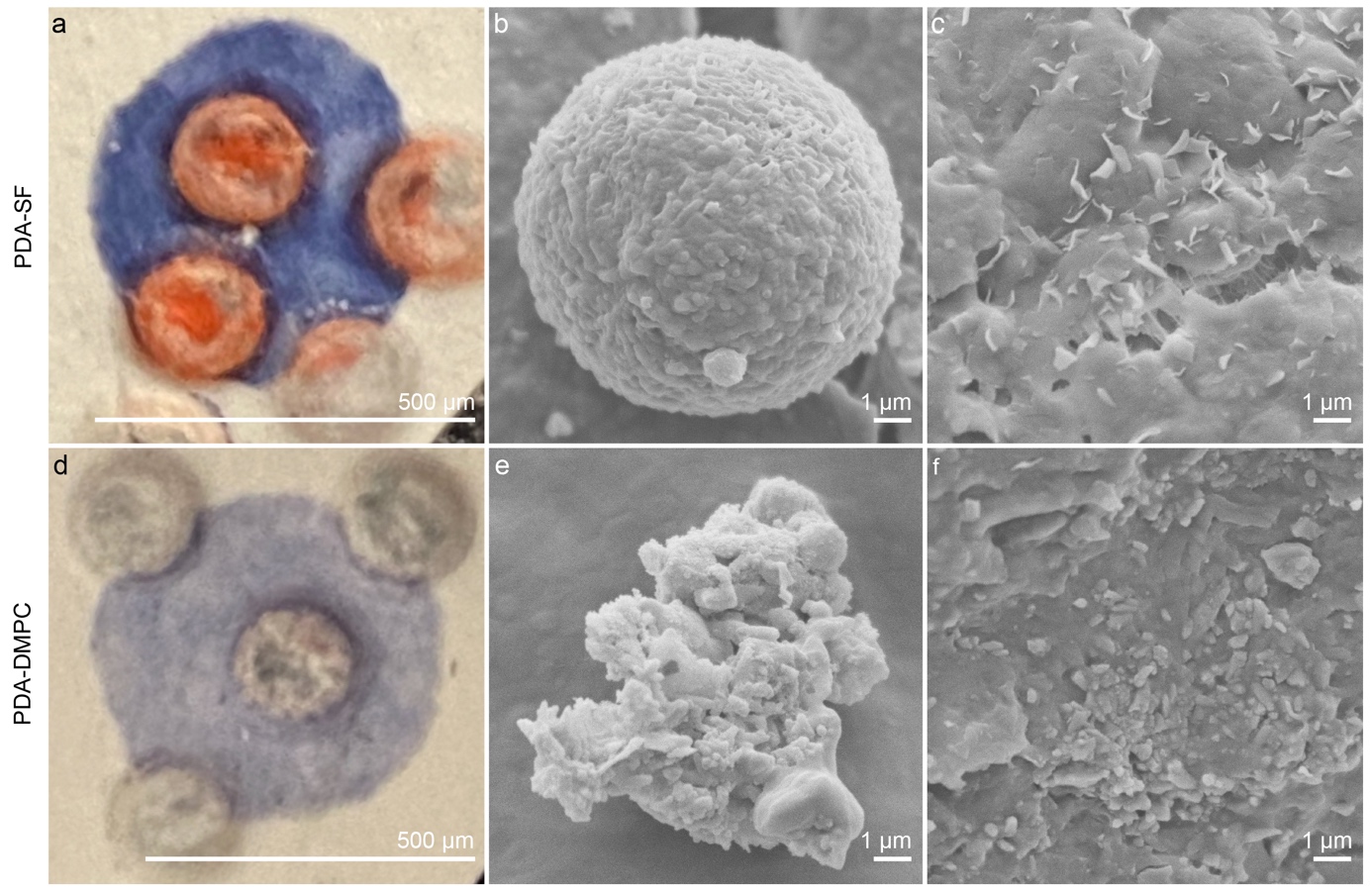


Figure S13. Morphology of the PDA-SF (top row) and PDA-DMPC (bottom row) vesicles cast on paper substrates before and after indentation. Macroscopic photographs of paper-based PDA-SF (a) and PDA-DMPC (d) sensors after mechanical activation. The indented regions display red (a) and grey (d) circular spots, respectively left by the dart. Corresponding SEM images of the PDA-SF vesicles (b, c) and of the PDA-DMPC vesicles (e, f) before (b, e) and after (c, f) indentation.


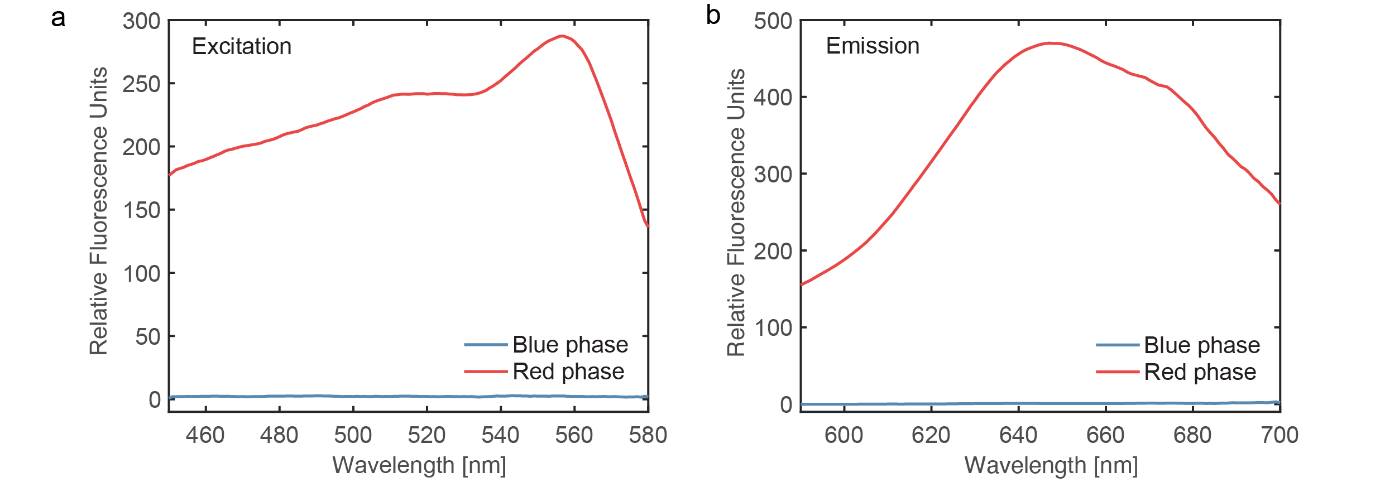


Figure S14. a. Excitation and b. emission spectra of the blue and red phase of PDA. The blue phase is not fluorescent, while the red phase shows a broadband absorption peak at λ_Abs_ = 556 nm, and an emission peak at λ_Em_ = 650 nm.


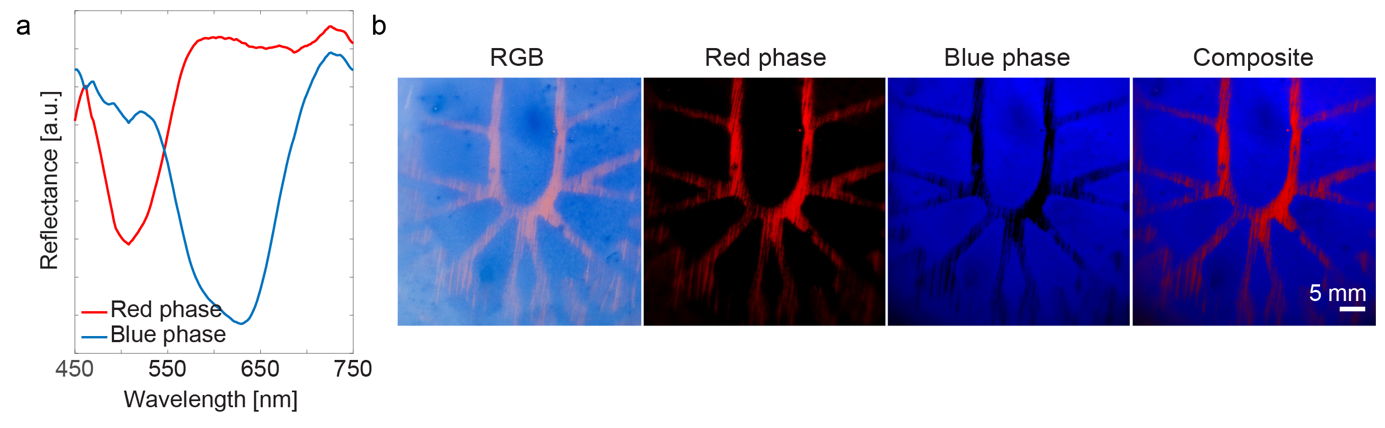


Figure S15. a. Normalized reflectance as a function of wavelength of the red and blue phase of PDA-SF sensor cast on a polystyrene substrate. b. Macroscopic (RGB), false-color individual spectral components (Red phase, Blue phase), and false-color reconstructed micrographs (Composite) of the mechanochromic PDA-SF film applied on a polystyrene substrate being pressed with a shoe with the vesicle’s layer facing the ground. The pattern of the sole is transferred on the mechanochromic sensor.
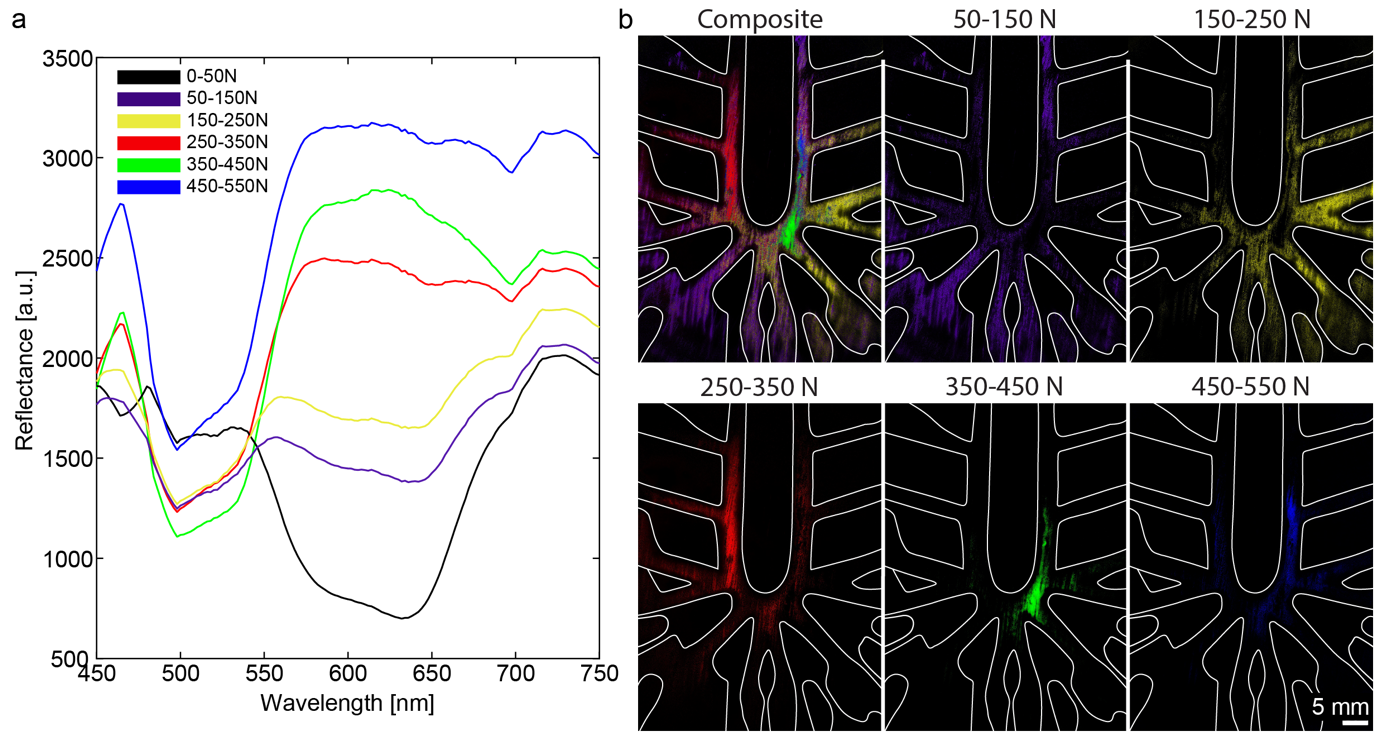


Figure S16. a. Calibrated reflectance spectra as a function of wavelength and b. corresponding calibrated false-color images of the mechanochromic PDA-SF film applied on a polystyrene substrate after being pressed with a shoe. The calibration for impact force is performed by evaluating the reflectance intensity at λ=600nm of the spectra reported in Figure 3 and by comparing them to the reflectance spectra collected from various portion of the sole pattern imprinted on the sensor.


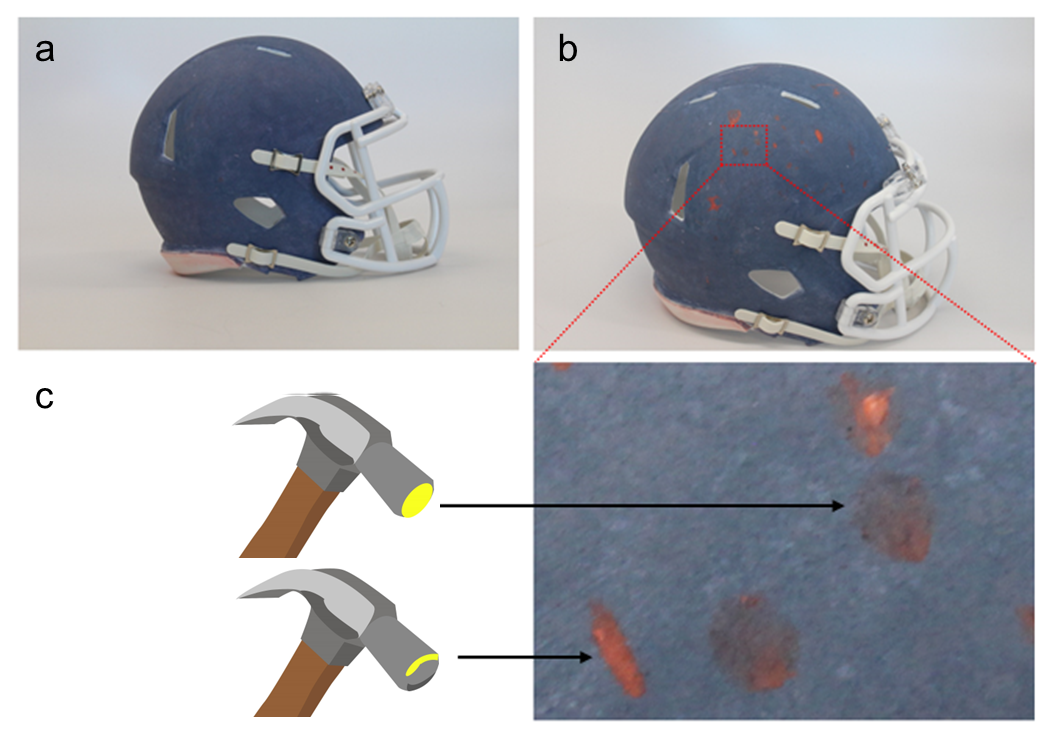


Figure S17. a. Photograph of the replica helmet before receiving the impact and b. after multiple hammer strikes. c. Close-up detail of the analyzed area using multispectral imaging, highlighting a flat impact and a cutting impact.

Table S1. Overview of the force and stress ranges reported for PDA-based mechanochromic sensors in the recent literature.

| System category | Force regime (indicative) | Stress regime (indicative) | Typical mechanical stimulus | Representative references |
| --- | --- | --- | --- | --- |
| **Nanoscale PDA mechanosensors** | 10–100 nN (lateral/normal AFM forces) | tip-localized, tens of MPa | AFM mechanochemistry, anisotropic PDA films | Zheng *et al.* 2024^56^; Zheng *et al.* 2025^31^; Das *et al.* 2024^57^ (nanoscale mode) |
| **Ultra-weak / weak forces** | 0.0004–0.49 N (compression); 0.006–0.08 N (friction) | 3.9–4.9 kPa (compression) | Ultra-soft compression on paper, weak sliding friction, calligraphy | Nakamitsu *et al.* 2021^28^; Shioda *et al.* 2023^30^ |
| **Weak–moderate friction / soft compression** | 0.2–31 N (friction, brushing, writing); – (compression only) | 1.23–675 kPa; ~1.7–5.1 MPa; 0.11–0.35 MPa | Toothbrushing, writing pressure, packaging scratches, soft compression mapping | Watanabe *et al.* 2020^29^; Terada *et al.* 2018^26^; Ono *et al.* 2022^58^; Das *et al.* 2024^57^ (macroscale mode) |
| **This work: PDA–SF core–shell vesicles (impact regime)** | 100–770 N | ~100–1000 MPa (1 mm impactor) | High-impact events: stomping, hammer blows, helmet impacts, drum strikes | This study |

Table S2. Correlation between UV activation times and UV dose delivered on the sensor’s surface.

| Activation time [s] | UV dose  [mJcm^-2^] | Activation time [min] | UV dose  [mJcm^-2^] |
| --- | --- | --- | --- |
| 0 | 0 | 2 | 300 |
| 5 | 12.5 | 5 | 750 |
| 10 | 25 | 10 | 1500 |
| 20 | 50 | 60 | 9000 |
| 60 | 150 | 180 | 27000 |

Table S3. Calculated values for velocity, energy, and impact force delivered by the dart falling from different heights.

| Falling height [cm] | Falling velocity [ms^-1^] | Kinetic energy [J] | Impact force [N] deformation 0.3 mm |
| --- | --- | --- | --- |
| 2 | 0.62 | 0.088 | 110 |
| 3 | 0.76 | 0.13 | 220 |
| 4 | 0.88 | 0.176 | 330 |
| 5 | 0.99 | 0.22 | 440 |
